# Effect of Nb Content on Tribocorrosion Behavior of TiZrNbTa Refractory Multi-Principal Element Alloys in Sodium Chloride Solution

**DOI:** 10.3390/ma19214727

**Published:** 2026-11-06

**Authors:** Zening Wang, Yi Zhang, Jinping Wu, Yu Yan, Lijie Qiao

**Affiliations:** Beijing Advanced Innovation Center for Materials Genome Engineering, Corrosion and Protection Center, Institute for Advanced Materials and Technology, University of Science and Technology Beijing, Beijing 100083, China; wangzeningning@163.com (Z.W.);

**Keywords:** multi-principal element alloys, tribocorrosion, wear–corrosion synergy, XPS

## Abstract

In this work, the effect of Nb content on the microstructure, passive film stability, and tribocorrosion behavior of TiZrNbTa refractory multi-principal element alloys (RMPEAs) was evaluated in 0.9 wt.% NaCl solution. Three designed alloys, Ti_45_Zr_45_Nb_5_Ta_5_, Ti_35_Zr_35_Nb_25_Ta_5_ and Ti_31.67_Zr_31.67_Nb_31.67_Ta_5_, were examined via electrochemical measurements, reciprocating sliding tests, X-ray photoelectron spectroscopy and surface/subsurface characterization. All alloys exhibited a single-phase body-centered cubic (BCC) solid solution. Increasing Nb refined dendritic grains but aggravated elemental segregation and reduced hardness from 320 ± 10 HV to 230 ± 10 HV. Reductions in corrosion and passive current densities demonstrated improved corrosion resistance and passive film stability. However, repassivation kinetics deteriorated because reduced Ti and Zr availability limited their preferential oxidation, which governed rapid passive film formation on fresh surfaces. Accordingly, higher Nb did not yield superior tribocorrosion resistance. The wear mechanism shifted from mild abrasive wear with continuous tribofilms toward severe ploughing, plastic deformation, delamination and microcracking. Under open-circuit potential (OCP) conditions, the average friction coefficient increased from 0.51 ± 0.05 to 0.78 ± 0.08, and the tribocorrosion rate rose from (3.75 ± 0.11) × 10^−3^ to (10.27 ± 0.30) × 10^−3^ mm^3^·N^−1^·m^−1^. Pure mechanical wear accounted for 70.72–79.11% of total material loss, and corrosion-accelerated wear dominated the synergistic component. The degraded performance at high Nb contents arose from the combined influence of decreased hardness, severe segregation, pit-triggered crack growth and suppressed surface work hardening. All of these factors amplified corrosion-accelerated wear. These findings revealed the dual function of Nb in TiZrNbTa RMPEAs. Nb stabilized the passive film while exacerbating tribocorrosion damage.

## 1. Introduction

Multi-principal element alloys (MPEAs), also known as high-entropy alloys (HEAs), open up an entirely new alloy design space. Their high configurational entropy, severe lattice distortion, sluggish diffusion and cocktail effect endow the alloys with unique phase stability, together with mechanical and chemical properties that are generally superior to those of conventional dilute solid solutions. Senkov et al. [1,2] proposed refractory multi-principal element alloys (RMPEAs) with refractory elements (Mo, Nb, Ta, W, V, Hf, Zr, Ti) as principal constituents. Featuring high melting points, excellent high-temperature strength retention and high hardness, RMPEAs serve as candidate materials for extreme service environments. Furthermore, the favorable biocompatibility [3] and strong oxygen affinity [4] of their constituent elements yield outstanding passivation performance, granting RMPEAs great application potential in biomedical implant materials.

In certain service environments, material surfaces are subjected to both environmental corrosion and friction. The interplay between these two effects accelerates material degradation and failure [5,6]. MPEAs exhibit a broad tunability in surface properties such as passivation and oxidation resistance, rendering them highly appealing for tribocorrosion applications [7]. However, current research on the tribocorrosion behavior of MPEAs remains limited. Most existing studies focus on the mechanisms under individual corrosion or wear conditions, while the material damage mechanism under their coupled interaction remains unclear. Yang et al. [8] investigated the damage behavior of CoCrFeNi-X (Ti, Mn, Mo, Al) HEA coatings under reciprocating friction in a 3.5 wt.% NaCl solution. They found that friction increases the corrosion rate within the worn area by 2–3 orders of magnitude. Although corrosion products can reduce the friction coefficient, localized damage induced by corrosion significantly increases the wear volume, and corrosion-triggered wear serves as the primary contributor to material loss. Diao et al. [9] investigated the tribocorrosion mechanisms of CoCrFeNiTi_x_ alloys in simulated seawater. Obvious corrosion-promoted wear appears in low-Ti alloys. For high-Ti alloys, corrosion of the R phase (HCP structure similar to Ni_3_Fe) induces delamination of the Laves phase and degrades tribocorrosion performance. The Ti_0.3_ alloy achieves the optimal tribocorrosion resistance owing to the anodic protection provided by the R phase. Wang et al. [10] quantitatively characterized the corrosion wear synergistic behaviors of FeCrNiCo(I-0), FeCrNiCoAl(I-Al) and FeCrNiCoMo(I-Mo) HEAs in artificial seawater. Preferential Al dissolution in the I-Al alloy yields a low friction coefficient and high wear rate. Molybdate-containing wear debris fills FCC-phase corrosion pits and gives the I-Mo alloy the lowest wear rate. Wear dominates tribocorrosion damage and the rise in corrosion rate induced by wear is 100 times that of the wear rate increase triggered by corrosion. Zhang et al. [11] investigated the tribocorrosion behavior of CoCrFeNiCu_0.3_ HEAs with different Cu distribution states in 3.5 wt.% NaCl solution. Homogeneous Cu distribution suppresses corrosive microcouples and promotes the formation of protective chromium oxide layers. It transforms the tribocorrosion mechanism from abrasive and corrosive wear to mild abrasive and oxidative wear and achieves optimal tribocorrosion resistance. Chen et al. [12] studied the repassivation behavior of Al_0.1_CrCoFeNi MPEAs during tribocorrosion. They found that selective oxidation of Cr, Co and Fe during repassivation leads to Ni enrichment on the alloy surface. The increased Ni content raises the electron work function and reduces surface reactivity. It further inhibits the repassivation process after the passive film is damaged by pitting or tribocorrosion. These studies indicate that most studies on the tribocorrosion behavior of MPEAs focus on 3d transition metal alloy systems represented by CoCrFeNi. Existing studies have shown that there is a significant coupling interaction between friction and corrosion in MPEAs. On one hand, the type and content of alloy elements regulate the competition and synergy between wear and corrosion by changing phase composition and microstructure. On the other hand, the passivation behavior of alloys including the stability of passive films and their repassivation ability after damage directly affects the evolution of tribocorrosion damage and material loss rate.

Current studies on RMPEAs mainly focus on their corrosion resistance and corrosion mechanisms. Research on their tribocorrosion behavior especially the coupled interaction between wear and corrosion remains scarce. Zeng et al. [13] studied the tribocorrosion behavior of Ti_x_NbCrMoAl RHEAs with varied Ti contents in molten aluminum and found their better tribocorrosion resistance than conventional materials. This improvement arises from reduced alloy reactivity with molten aluminum and hard corrosion products, while material failure is controlled by synergistic corrosion wear interplay of matrix corrosion and wear triggered spalling of corrosion products. Ling et al. [14] fabricated TiNbCr RMPEAs and investigated the effect of Ti/Nb atomic ratio on their tribocorrosion behavior in molten aluminum. The alloy with 40 at.% Nb retains the BCC structure and forms a triple-synergistic interfacial armoring mechanism. Ti_48_Nb_40_Cr_12_ exhibits much lower volume loss than TC4 and H13 steel under molten aluminum tribocorrosion. Xiao et al. [15] alloyed TiZrHfNb-based RMPEAs with Ta and Mo to enhance their corrosion and wear resistance via passivation film formation and hardness improvement. Their work reveals coupled electrochemical and mechanical interactions during tribocorrosion. Tribocorrosion degradation under anodic potential is marginally higher than at open- circuit potential (OCP), and the TiZrHfNb_1.83_Mo_1.83_ alloy achieves the optimal tribocorrosion performance. It can be seen that the composition and ratio of alloying elements exert a significant regulatory effect on the tribocorrosion behavior of RMPEAs. By altering the phase composition of alloys, and the characteristics of surface passivation or corrosion product films, the coupled mechanism of corrosion and wear can be effectively modified, which further determines the evolution law of tribocorrosion damage of alloys. In the TiZrNbTa system, Nb possesses a relatively high elastic modulus and melting point among the constituent elements. Adjusting Nb content can directly modulate the valence electron concentration, lattice distortion degree, solid solution strengthening level and the strength–plasticity balance of the BCC matrix [16,17]. On the other hand, variations in Nb content may also alter the composition and stability of surface oxide films, thereby affecting the wear behavior of alloys [18,19,20]. Nevertheless, systematic research linking Nb alloying extent to the tribocorrosion behavior of TiZrNbTa RMPEAs remains scarce to date, and the underlying damage mechanism as well as the tribocorrosion interaction are still unclear.

In this work, three TiZrNbTa RMPEAs with controlled Nb content (Ti_45_Zr_45_Nb_5_Ta_5_, Ti_35_Zr_35_Nb_25_Ta_5_ and Ti_31.67_Zr_31.67_Nb_31.67_Ta_5_) were fabricated via arc melting. The microstructure, phase composition, and hardness of the alloys were systematically characterized. Their tribocorrosion behavior was evaluated in NaCl solution and the synergistic effect of friction and corrosion was quantified to distinguish material loss caused by mechanical wear and electrochemical corrosion. By correlating Nb content with microstructure characteristics mechanical response surface film properties and damage morphology, the mechanism of Nb content affecting alloy tribocorrosion was clarified, which provides theoretical guidance for the design of RMPEAs.

## 2. Materials and Methods

### 2.1. Material Preparation

Commercial pure Ti, Nb, Ta and Zr metals (all with purity of 99.9 wt.%, Beijing General Research Institute for Nonferrous Metals, Beijing, China) were used as raw materials in this study. RMPEAs were fabricated via arc melting in a water-cooled copper crucible under an argon atmosphere. To ensure compositional homogeneity, the molten alloy was remelted five times, followed by casting into a copper mold to obtain alloy ingots with uniform compositions. In this work, compositional homogenization was achieved solely by multiple arc remelting, and no subsequent homogenization heat treatment was performed on the as-cast alloys. All characterization tests in this study were carried out on the above-mentioned as-cast alloy specimens. Each ingot was cut into square sheets of 15.00 ± 0.05 mm × 5.00 ± 0.05 mm × 1.00 ± 0.02 mm by wire electrical discharge machining. The specimen surfaces were sequentially ground using silicon carbide abrasive papers with grit sizes of 1000, 2000, 3000 and 5000 (Yingkou Sanzheng New Technology Industry Co., Ltd., Yingkou, China).

### 2.2. Microstructure Characterization

The phase constitution of the alloys was determined by X-ray diffraction (XRD, D8 Advance, Bruker, Karlsruhe, Germany) using Cu-Kα radiation. XRD patterns were collected over a 2*θ* range at a scanning speed of 5°·min^−1^. Specimens for microstructural observation were cut from the as-cast ingots, sequentially ground with silicon carbide abrasive papers up to 5000 grit, and then polished with 0.04 μm diamond suspension (Truer, Suzhou, China), followed by ultrasonic cleaning in ethanol and warm-air drying. Back-scattered electron (BSE) imaging and energy-dispersive X-ray spectroscopy (EDS) were performed on a Zeiss SUPRA 55 field-emission scanning electron microscope (FE-SEM, Carl Zeiss AG, Oberkochen, Germany) at an accelerating voltage of 15 kV and a working distance of approximately 10 mm. EDS elemental mapping was acquired at 15 kV with a pixel dwell time of 100 μs and a map resolution of 1024 × 768 pixels. For quantitative compositional analysis, five point spectra were acquired in each of the dendritic and interdendritic regions with an acquisition time of 60 s per spectrum, and the results were quantified using the built-in ZAF (atomic number, absorption and fluorescence) correction without external standards. The characterization of the initial as-cast microstructure was carried out independently of the worn-track characterization after subsequent tribocorrosion tests.

### 2.3. Microhardness Testing

Microhardness tests of all alloy specimens were performed using a high-precision digital Vickers hardness tester (HVS-1000Z, Shanghai Caikon Optical Instrument Co., Ltd., Shanghai, China, load accuracy ± 0.5%). A programmed automatic loading dwell and unloading sequence was adopted for the test. The experimental parameters were set as follows: an applied load of 0.01 kg (10 gf, approximately 98.07 mN), a loading rate of 50 μm·s^−1^, and a dwell time of 15 s, followed by a 2 s stabilization period after unloading to obtain high-quality indentation images. Ten measurements were carried out at randomly selected areas on the surface of each specimen, and the arithmetic mean and standard deviation were calculated for the results.

### 2.4. Electrochemical Measurements

Electrochemical tests were performed in a standard three-electrode electrolytic cell. A saturated calomel electrode (SCE, Model 232, Shanghai INESA Scientific Instrument Co., Ltd., Shanghai, China) was used as the reference electrode, and a platinum sheet served as the counter electrode (Wuhan Corrtest Instruments Corp., Ltd., Wuhan, China). All electrochemical measurements were conducted with an Ivium Vertex electrochemical workstation (Ivium Technologies B.V., Eindhoven, The Netherlands) in 0.9 wt.% NaCl solution (sodium chloride, Sinopharm Chemical Reagent Co., Ltd., Shanghai, China) at room temperature (25 ± 2 °C) under atmospheric pressure. Although 0.5 M NaCl is commonly used in static corrosion studies, 0.9 wt.% NaCl (approximately 0.154 M) was selected to approximate physiological saline and provide a relevant medium for tribocorrosion under reciprocating sliding. This choice is particularly relevant to biomedical implants and components operating in mild saline environments, rather than the more aggressive high-chloride conditions encountered in marine applications. After connecting copper wires (Aladdin, Shanghai, China), the specimens were encapsulated with epoxy resin, leaving only the working surface (15 mm × 5 mm, i.e., 75 mm^2^) exposed. The edges and back side were fully coated with epoxy resin (Shanghai Meixin Resin Co., Ltd., Shanghai, China), and after curing the encapsulation was examined under an optical microscope to confirm that no metal was exposed, so that the exposed area was accurately controlled and used for current-density normalization. The specimens were then ground, alcohol-degreased (anhydrous ethanol, Sinopharm Chemical Reagent Co., Ltd., Shanghai, China), cleaned and warm-air-dried. Before testing, the working electrode was immersed in the electrolyte for 120 ± 2 min to allow the system potential to become relatively stable. Potentiodynamic polarization tests were performed under conditions with and without sliding contact. The potential scanning range was from 0.30 V negative to the corrosion potential (*E*_corr_) up to 3.0 V_SCE_, and the scanning rate was 1.0 mV·s^−1^. Although the scan rate used here is higher than lower steady-state-oriented values such as 0.16 mV·s^−1^, it is not expected to introduce a significant bias in the present comparative analysis of these strongly passivating TiZrNbTa RMPEAs [21,22]. Nevertheless, an appropriate potential scan rate is important for minimizing distortion in Tafel-slope and corrosion current density (*i*_corr_) analyses [21,22]. In addition, the OCP and cathodic polarization curves of all samples were monitored under conditions with and without sliding contact. For each alloy, electrochemical measurements were performed on three independently prepared specimens, and all electrochemical parameters are reported as the mean and standard deviation.

### 2.5. Tribocorrosion Tests and Characterization

Tribocorrosion tests were carried out on the samples under reciprocating sliding conditions in 0.9 wt.% NaCl solution (identical to that used in the electrochemical measurements) using a tribometer (UMT-2, CETR Inc., Campbell, CA, USA) equipped with a linear reciprocating drive module and a three-electrode electrochemical cell. This custom-modified tribometer with a liquid corrosion-cell module provides precise normal-load and sliding-motion control (1 mN load resolution, 0.1 μm displacement resolution). Further equipment details are available in the reference [23]. During testing, a closed-loop-controlled precision loading stage maintains normal load. A high-resolution load cell records friction force for real-time calculation of the friction coefficient. A displacement-controlled linear drive defines reciprocating stroke and sliding frequency. The counterbody ball sits perpendicularly to specimens on the friction sensor. Its integrated electrochemical cell links to an Ivium Vertex workstation to collect open-circuit potential, current density and COF throughout sliding tests. The counterbody was an alumina (Al_2_O_3_) ball with a diameter of 10.00 ± 0.05 mm. The cyclic sliding stroke was 30.00 ± 0.05 mm at a frequency of 1.0 Hz, corresponding to a sliding speed of 0.06 ± 0.0001 m·s^−1^. The applied normal load was 1.0 N, and the maximum Hertz contact pressures were 288.5 ± 2.0 MPa, 321.6 ± 2.2 MPa and 336.8 ± 2.3 MPa for Ti_45_Zr_45_Nb_5_Ta_5_, Ti_35_Zr_35_Nb_25_Ta_5_ and Ti_31.67_Zr_31.67_Nb_31.67_Ta_5_ alloys, respectively. Tribocorrosion measurements were performed at the OCP and a cathodic potential of −0.8 V_SCE_, with a friction duration of 1000 ± 2 s for both conditions. The tribocorrosion tests at the cathodic potential were used to obtain the pure wear rate, namely the purely mechanical wear. The evolution of OCP, cathodic and anodic current densities as well as the corresponding coefficient of friction (COF) were recorded before, during and after sliding. Each tribocorrosion test was repeated on three independently prepared specimens for every alloy. The friction coefficients, wear rates and characteristic electrochemical quantities are reported as the mean and standard deviation. Ten Vickers microhardness measurements were performed at different regions of the wear track after the tribological test. In contrast to the initial microstructure characterization described in Section 2.2, SEM, EDS and a 3D profilometer (LEXT OLS5000, Olympus Corporation, Hachioji, Tokyo, Japan) were then employed to characterize the morphology, chemical composition and three-dimensional topography of the wear tracks after tribocorrosion tests.

### 2.6. Characterization of Passive Film Composition

X-ray photoelectron spectroscopy (XPS, PHI Quantera SXM, Physical Electronics, Chanhassen, MN, USA) was utilized to investigate the chemical composition of passive films generated on the alloy surfaces. A monochromatic Al-Kα X-ray source (h_ν_ = 1486.6 eV) was adopted for excitation. During XPS characterization, the photoelectron take-off angle was maintained at 45°, and the analysis area was a 100 μm spot. Survey spectra were recorded across a binding energy interval of 1200 eV, with the pass energy set to 55.0 eV and a spectral resolution of 0.1 eV.

## 3. Results

### 3.1. Phase Composition and Microstructure

**Figure 1:**
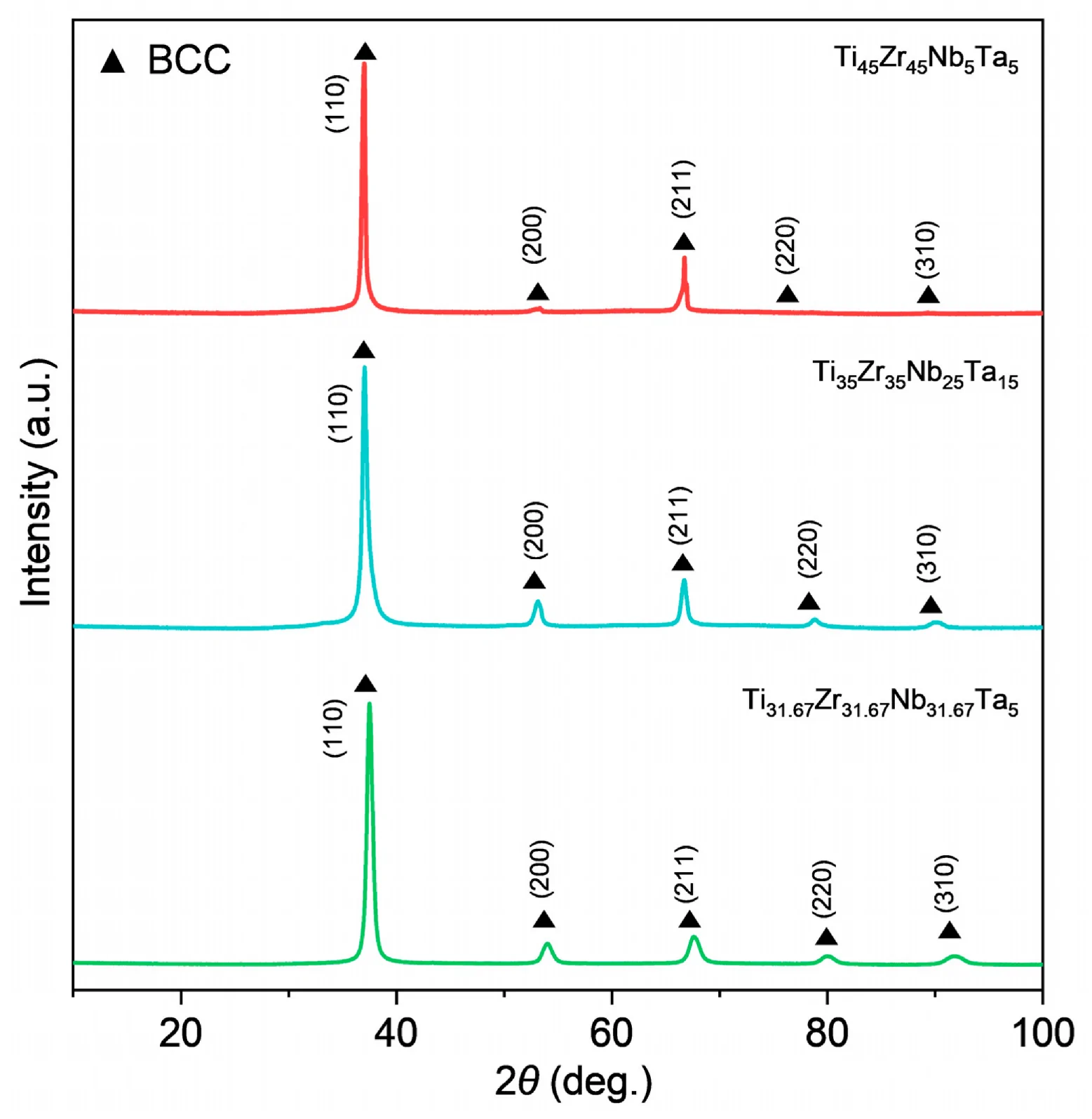
XRD patterns of all investigated samples.

Figure 1 shows the XRD patterns of as-cast Ti_45_Zr_45_Nb_5_Ta_5_, Ti_35_Zr_35_Nb_25_Ta_5_ and Ti_31.67_Zr_31.67_Nb_31.67_Ta_5_ alloys. For each alloy, the XRD measurement was repeated three times at different positions on the specimen surface, and the patterns were essentially identical, confirming the reproducibility of the phase constitution. Careful indexing of diffraction peaks confirms that these peaks correspond to the (110), (200), (211), (220) and (310) crystal planes of the body-centered cubic (BCC) structure. The diffraction peaks lie close to, and at the low-angle side of, the reference peak positions of bcc β-Ti (JCPDS No. 44-1288) and bcc Nb (JCPDS No. 35-0789), which is consistent with previously reported TiZrNbTa RMPEAs in reference [24]. Quantitative analysis based on Gaussian peak fitting yields lattice parameters of a = 3.435 ± 0.005 Å, 3.431 ± 0.002 Å and 3.391 ± 0.004 Å for the three alloys, respectively. As the Nb content increases from 5 at.% to 31.67 at.%, all diffraction peaks shift toward higher Bragg angles, approaching the bcc Nb-rich reference position. Taking the (110) reflection as an example, its position moves from 36.96° to 37.42°, indicating a gradual reduction in the lattice parameter of TiZrNbTa RMPEAs. Such peak shift originates from lattice contraction, which is associated with the decreased content of Zr (with the largest atomic radius) and the increased proportion of smaller Nb atoms [25,26]. In addition, no extra diffraction peaks originating from intermetallic compounds or precipitated phases are detected in the XRD patterns of the three TiZrNbTa RMPEAs. All three alloys maintain a single-phase BCC solid solution without secondary phases, demonstrating high phase stability of the TiZrNbTa system within the compositional range investigated in this work.

**Figure 2:**
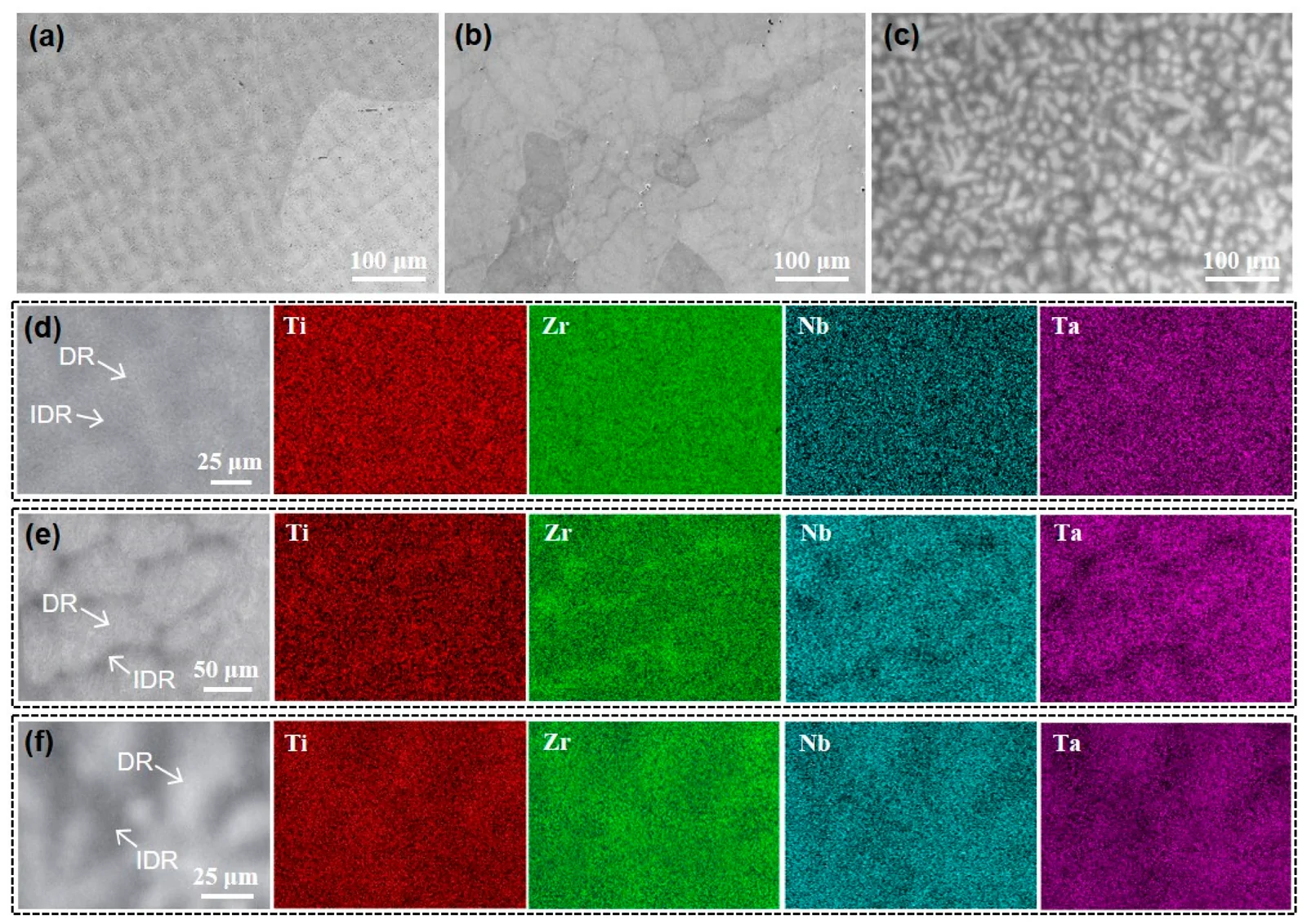
SEM-BSE images and corresponding EDS elemental mapping of as-cast TiZrNbTa RMPEAs: (**a**) Ti_45_Zr_45_Nb_5_Ta_5_; (**b**) Ti_35_Zr_35_Nb_25_Ta_5_; (**c**) Ti_31.67_Zr_31.67_Nb_31.67_Ta_5_; (**d**–**f**) EDS maps of Ti, Zr, Nb and Ta for alloys in (**a**–**c**), respectively.

The microstructures of as-cast Ti_45_Zr_45_Nb_5_Ta_5_, Ti_35_Zr_35_Nb_25_Ta_5_ and Ti_31.67_Zr_31.67_Nb_31.67_Ta_5_ alloys are shown in Figure 2a–c. All three alloys exhibit typical cast dendritic structures with clear grain boundaries, and the grain size decreases with increasing Nb content. Here, the dendritic region (DR) refers to the primary dendrite arms and trunks that solidify first during cooling, whereas the interdendritic region (IDR) refers to the last-solidified areas located between neighboring dendrite arms. Ti_45_Zr_45_Nb_5_Ta_5_ shows regular herringbone dendrites, while Ti_35_Zr_35_Nb_25_Ta_5_ and Ti_31.67_Zr_31.67_Nb_31.67_Ta_5_ display dendritic morphology, and the dendritic arms are continuously refined as Nb content rises. This phenomenon originates from the low diffusion rate of Nb, which exerts a strong pinning effect on grain boundaries and restrains grain growth during solidification and cooling. The light-gray dendritic regions and dark-gray interdendritic regions in the back-scattered electron (BSE) images (as marked and distinguished in the BSE micrographs of Figure 2d–f) reflect solute redistribution during solidification. Nb and Ta possess higher atomic numbers than Ti and Zr, so the Nb/Ta-rich dendritic regions exhibit brighter contrast, while the Ti/Zr-rich interdendritic regions show darker contrast. Combined with EDS mapping quantitative results in Figure 2d–f and Table 1, the dendritic regions are enriched in Nb and Ta, whereas interdendritic regions possess higher concentrations of Ti and Zr [27]. All compositional values in Table 1 are reported as the mean together with its corresponding standard deviation. These data are derived from five EDS point analyses conducted at randomly selected positions within each region. The degree of elemental segregation increases significantly with rising Nb content. The composition of Ti_45_Zr_45_Nb_5_Ta_5_ is close to the TiZr quasi-binary system, which features similar atomic sizes, near-zero enthalpy of mixing and a partition coefficient close to unity for each element, leading to weak solute redistribution. As Nb content increases, high-melting-point Nb and Ta preferentially solidify into the solid phase, and Ti and Zr are rejected into the residual liquid and enriched in interdendritic areas [27,28]. The positive enthalpy of mixing and the large atomic size mismatch between Nb or Ta and Zr cause the solid phase to repel large-sized Zr atoms, thereby resulting in a partition coefficient of less than 1. Under non-equilibrium solidification, Zr continuously accumulates in the residual liquid and further intensifies interdendritic segregation [29]. Since Zr has a higher melting point than Ti, its interdendritic enrichment cannot be merely explained by melting point difference; it is mainly governed by thermodynamic and atomic size effects [29]. This indicates that increased Nb alloying is the primary cause for aggravated segregation. XRD results confirm that all three alloys maintain a single-phase BCC solid solution without intermetallic compounds or secondary phases. Within this compositional range, elemental segregation only induces local compositional fluctuation and lattice distortion in the solid solution, without altering the crystallographic symmetry of the matrix.

### 3.2. Potentiodynamic Polarization

**Figure 3:**
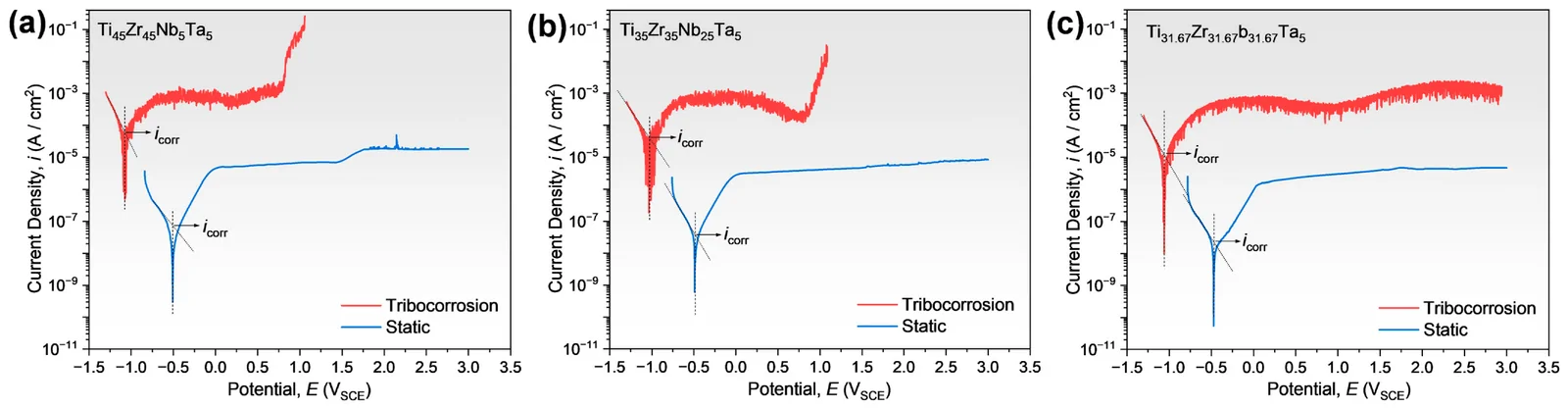
PDP curves of TiZrNbTa RMPEAs tested under static and tribocorrosion conditions: (**a**) Ti_45_Zr_45_Nb_5_Ta_5_; (**b**) Ti_35_Zr_35_Nb_25_Ta_5_; (**c**) Ti_31.67_Zr_31.67_Nb_31.67_Ta_5_.

Figure 3 presents the potentiodynamic polarization (PDP) curves of all tested alloys under static and tribocorrosion conditions. All PDP curves pass directly from the Tafel region into a wide passive region, with no active dissolution peak observed, indicating that the alloys exhibit typical self-passivation behavior at the *E*_corr_ [30]. All electrochemical parameters were extracted from polarization curves using a unified procedure. The *i*_corr_ under both static and tribocorrosion conditions was acquired by fitting the cathodic Tafel line within the range of approximately 100 mV to 50 mV below the *E*_corr_ and extrapolating this line to *E*_corr_. The anodic branch was excluded from fitting, as spontaneous passivation inhibits the formation of a Tafel region on the anodic side. This single-branch fitting method is a standard treatment for passivating metallic systems and has been validated through independent measurements on a wide range of passivating metallic systems [31,32,33]. For static tests, the *i*_pass_ was defined as the average current density over the potential window from 0 V_SCE_ to 1.0 V_SCE_. Under tribocorrosion conditions, ipass was taken as the average current density over the potential window from −0.75 V_SCE_ to 0.5 V_SCE_. The *i*_pass_ of the Ti_45_Zr_45_Nb_5_Ta_5_, Ti_35_Zr_35_Nb_25_Ta_5_, and Ti_31.67_Zr_31.67_Nb_31.67_Ta_5_ alloys are approximately 6.95 ± 0.83, 4.27 ± 0.78, and 3.93 ± 0.75 μA·cm^−2^, respectively. As the potential increases, a pronounced current rise emerges at approximately 1.25 V_SCE_, which mainly originates from the oxygen evolution reaction (OER) triggered by electron transfer across the surface oxide film [34,35]. In contrast, the magnitude of this current rise decreases with increasing Nb content, indicating that the passive film possesses enhanced dielectric properties and can effectively block electron transfer. Additionally, this behavior is also associated with the increased activation energy and reduced diffusivity of oxygen vacancies within the passive film [36]. This current rise mainly originates from the OER, while the chlorine evolution reaction (CER) exerts only minor effects. In near-neutral NaCl solution, the O_2_/H_2_O equilibrium potential (~0.82 V_SHE_) is far lower than the measured onset potential (1.49 V_SHE_), and this difference reflects the high OER overpotential on the passive film. Although the equilibrium potential of CER in a 0.154 M chloride medium (1.41 V_SHE_) is only slightly lower than this onset potential, the kinetics of CER are substantially inhibited on valve-metal passive films (e.g., TiO_2_ exhibits nearly no catalytic activity toward anodic chlorine evolution [37,38,39,40]). As the Nb content increases, the current amplitude gradually decreases. This trend is consistent with improved dielectric properties and reduced oxygen vacancy diffusivity of the passive film, demonstrating that the process is film-controlled and dominated by electron transport within the oxide layer. In contrast, CER depends on chloride activity and interfacial adsorption rather than the bulk properties of the passive film [38,39]. Admittedly, the contribution of minor chloride oxidation cannot be fully ruled out within the narrow potential window between the CER equilibrium potential and the onset potential. Furthermore, chemical and structural evolution of the passive film should also be considered. For instance, the growth of crystalline oxides inside the oxide film with increasing potential may accelerate the OER [41,42]. Further oxidation of constituent elements into higher-valence oxides may also slightly increase the anodic passive current density, yet such contributions remain limited. No obvious compositional-dependent differentiation of oxidation peak potentials is observed in this work, and the peak current does not rise with increasing Nb content. 

The measured *i*_corr_ values are 76 ± 5, 40 ± 6, and 27 ± 3 nA·cm^−2^, respectively, indicating that the dissolution rate of the passive film decreases with increasing Nb content. For the Ti_45_Zr_45_Nb_5_Ta_5_ and Ti_35_Zr_35_Nb_25_Ta_5_ alloys, obvious metastable pitting current peaks can be observed at approximately 2.0 V_SCE_. These metastable events are distinguished from stable passive film breakdown by their transient character. The current spikes decay back to the passive current level within seconds as the metastable pits repassivate. Stable pitting breakdown is identified by an abrupt sustained current increase at least one order of magnitude above the passive level that does not decay upon continued polarization [43]. Notably, no stable film breakdown is detected within the scanned potential range up to 3.0 V_SCE_. This result reveals the excellent stability of the passive films. Once friction is applied to the surface, the overall current density of the polarization curves increases, and the *E*_corr_ shifts markedly in the negative direction to approximately −1.0 V_SCE_ (Figure 3), with pronounced fluctuations in the overall current. This is attributed to the influence of stirring on the cathodic reaction at the electrode surface and the depassivation–repassivation process induced by friction [23,44]. Intense current oscillations in the anodic region arise from periodic depassivation–repassivation events synchronized with the reciprocating sliding cycle (1.0 Hz). Each sliding pass of the counterbody ruptures the passive film and triggers vigorous anodic dissolution. The current then gradually decays as the passive film reforms. The Ti_45_Zr_45_Nb_5_Ta_5_ alloy exhibits the largest oscillation amplitude, which decreases with rising Nb content. This confirms that such current fluctuations originate from the intrinsic competition between depassivation and repassivation of the material, instead of instrumental noise. Friction leads to a significant increase in *i*_corr_, rising to 57 ± 3, 26 ± 5, and 10 ± 5 μA·cm^−2^, respectively, while the *i*_pass_ increases to 0.73 ± 0.05, 0.64 ± 0.08, and 0.46 ± 0.06 mA·cm^−2^. Notably, for the Ti_45_Zr_45_Nb_5_Ta_5_ and Ti_35_Zr_35_Nb_25_Ta_5_ alloys under friction, the current continuously increases when the polarization potential reaches approximately 0.79 ± 0.07 V_SCE_ and 0.85 ± 0.05 V_SCE_, respectively, implying localized accelerated dissolution or breakdown of the passive film. In contrast, when the polarization potential reaches 3.0 V_SCE_, the passive current of the Ti_31.67_Zr_31.67_Nb_31.67_Ta_5_ alloy remains fluctuating within a relatively stable range, indicating that its passive film still possesses high protective capability under tribocorrosion conditions. 

### 3.3. Open-Circuit Potential and Cathodic Polarization

**Figure 4:**
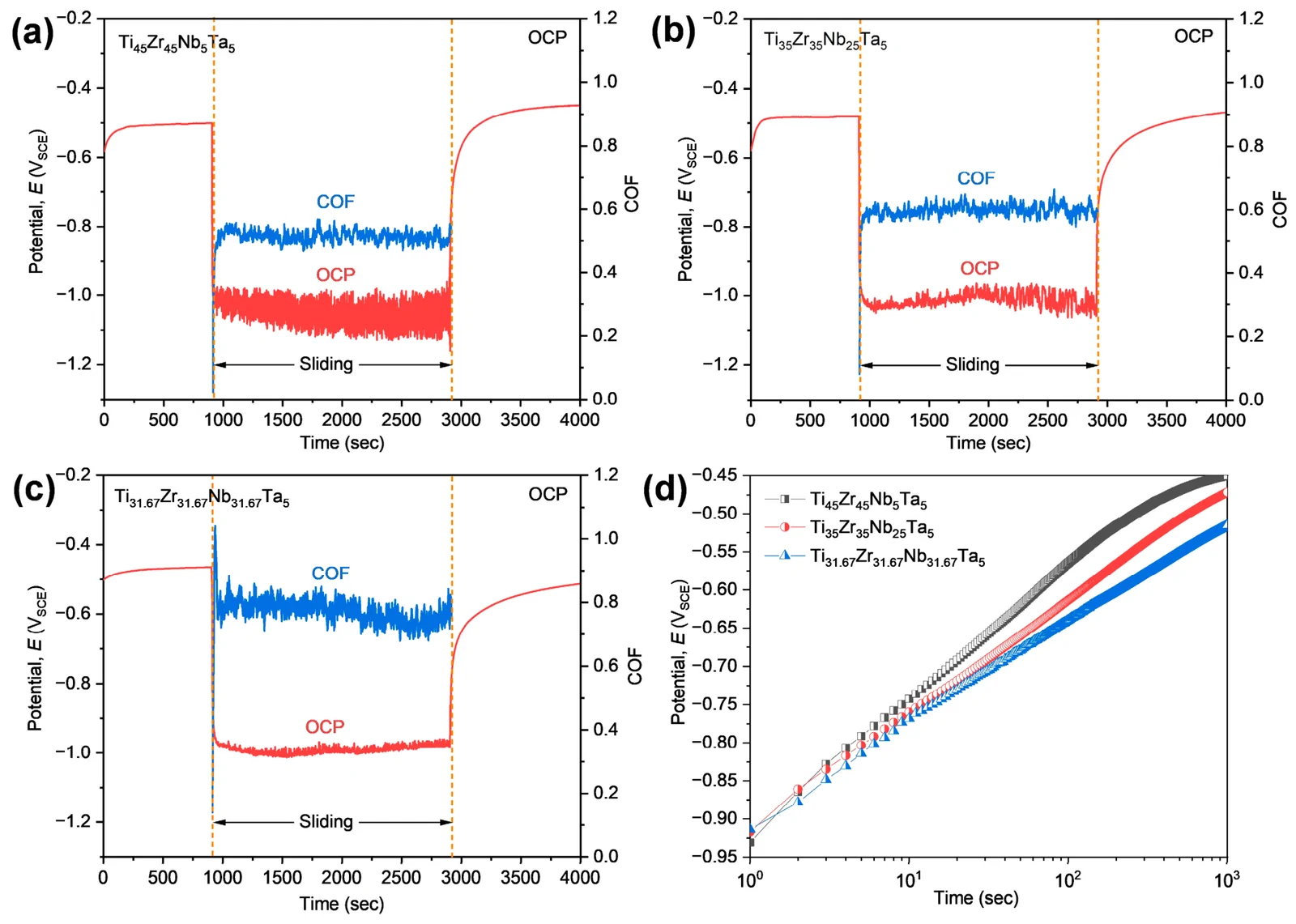
OCP and friction coefficient evolution of TiZrNbTa RMPEAs during tribocorrosion sliding: (**a**) Ti_45_Zr_45_Nb_5_Ta_5_; (**b**) Ti_35_Zr_35_Nb_25_Ta_5_; (**c**) Ti_31.67_Zr_31.67_Nb_31.67_Ta_5_; (**d**) OCP recovery curves after sliding in logarithmic time scale.

Figure 4 presents the in situ electrochemical response of the OCP of the alloys to tribocorrosion. After approximately 2 h of immersion, OCP data were first acquired for about 900 s, and then mechanical friction was applied to the sample surface. Upon initiation of friction, the OCP drops sharply, indicating mechanical removal of the surface passive film [45]. The initial OCP values of Ti_45_Zr_45_Nb_5_Ta_5_, Ti_35_Zr_35_Nb_25_Ta_5_, and Ti_31.67_Zr_31.67_Nb_31.67_Ta_5_ alloys measured before friction are approximately −0.51 ± 0.02, −0.48 ± 0.02, and −0.46 ± 0.03 V_SCE_, respectively. Friction renders the worn track anodic and the unfrictioned electrode area cathodic. Galvanic coupling between these two regions establishes a new electrode potential, which explains the OCP decrease under friction. The magnitude of the OCP reduction induced by friction for passive metals can be expressed as [46]:

(1) $$E_{c}\;={\;E}_{s}\;+\;a_{c}\;-\;b_{c}\log i_{a}-b_{c}\log\left[\left(\frac{\mathrm{LR}}{A_{c}}\right){\left(\frac{k_{\mathrm{wt}}}{0.003}\right)}^{0.333}\right]$$

where $E_{c}$ is the electrode potential after friction, i.e., the measured OCP value upon tribological loading. $i_{a}$ denotes the anodic dissolution current density within the worn track. $E_{s}$ is the measured OCP value before friction. $a_{c}$ and $b_{c}$ represent the anodic and cathodic Tafel slopes derived from static polarization curves, respectively. L and R are the worn track length and the radius of the counter ball, respectively. $k_{\mathrm{wt}}$ is the material wear rate, and $A_{c}$ corresponds to the cathode area. Therefore, the primary factors affecting the OCP drop of the material are the dissolution current in the anodic region, the cathode area, and the mechanical wear rate of the material. Subsequent analysis reveals that the mechanical wear rate of the materials follows the order: Ti_31.67_Zr_31.67_Nb_31.67_Ta_5_ > Ti_35_Zr_35_Nb_25_Ta_5_ > Ti_45_Zr_45_Nb_5_Ta_5_, while the cathode area exhibits the opposite trend. Nevertheless, the OCP reductions are 0.55 ± 0.07, 0.54 ± 0.05, and 0.52 ± 0.05 V, respectively. According to the above equation, this indicates that the anodic dissolution current generated on the fresh alloy surface during friction decreases in the sequence Ti_45_Zr_45_Nb_5_Ta_5_ > Ti_35_Zr_35_Nb_25_Ta_5_ > Ti_31.67_Zr_31.67_Nb_31.67_Ta_5_. This trend is consistent with the order of *i*_corr_ obtained from PDP tests under friction, meaning that the cation dissolution rate of the fresh alloy surface is suppressed with increasing Nb content. During friction, depassivation and repassivation processes on the material surface exist in dynamic equilibrium, causing the OCP to fluctuate around a certain potential level. The Ti_45_Zr_45_Nb_5_Ta_5_ alloy delivers the maximum OCP fluctuation amplitude. This amplitude decreases for the Ti_35_Zr_35_Nb_25_Ta_5_ alloy. The Ti_31.67_Zr_31.67_Nb_31.67_Ta_5_ alloy exhibits the weakest OCP fluctuation. The more pronounced OCP fluctuations reflect a stronger dynamic competition between friction-induced depassivation and surface repassivation, which is consistent with the rapid repassivation behavior of the Ti- and Zr-rich alloy. It therefore demonstrates that increasing the contents of Ti and Zr in the alloy is beneficial to its repassivation performance. It should be noted that each tribocorrosion test was repeated three times under identical conditions, and the results were highly reproducible. The characteristic quantities derived from Figure 4, including the OCP at the onset of friction, the magnitude of the friction-induced OCP drop, the OCP fluctuation amplitude, showed small scatter among the three parallel runs, confirming the reliability of the in situ electrochemical and friction data. When friction ceases, repassivation occurs and the OCP gradually recovers to a value close to its initial level. The rate of OCP rise during passivation recovery differs among the three alloys. Within a specified time period after the onset of repassivation, the repassivation rate can be quantitatively analyzed using the following formula [47]:

(2) $$\Delta E=k_{1}\log t\;+\;k_{2}$$

where ΔE is the amplitude of potential rise, *t* is the passivation recovery time, $k_{1}$ is the repassivation rate, and $k_{2}$ is a constant related to the solution. The value of $k_{2}$ is 0.1 for NaCl solution [47]. According to Equation (2), the linear region of OCP versus log *t* after the cessation of friction was fitted, yielding $k_{1}$ of 0.176 ± 0.011, 0.152 ± 0.015 and 0.129 ± 0.009 for the Ti_45_Zr_45_Nb_5_Ta_5_, Ti_35_Zr_35_Nb_25_Ta_5_, and Ti_31.67_Zr_31.67_Nb_31.67_Ta_5_ alloys, respectively. This indicates that a high cation dissolution rate on the bare metal surface facilitates the nucleation and growth of the passive film. 

**Figure 5:**
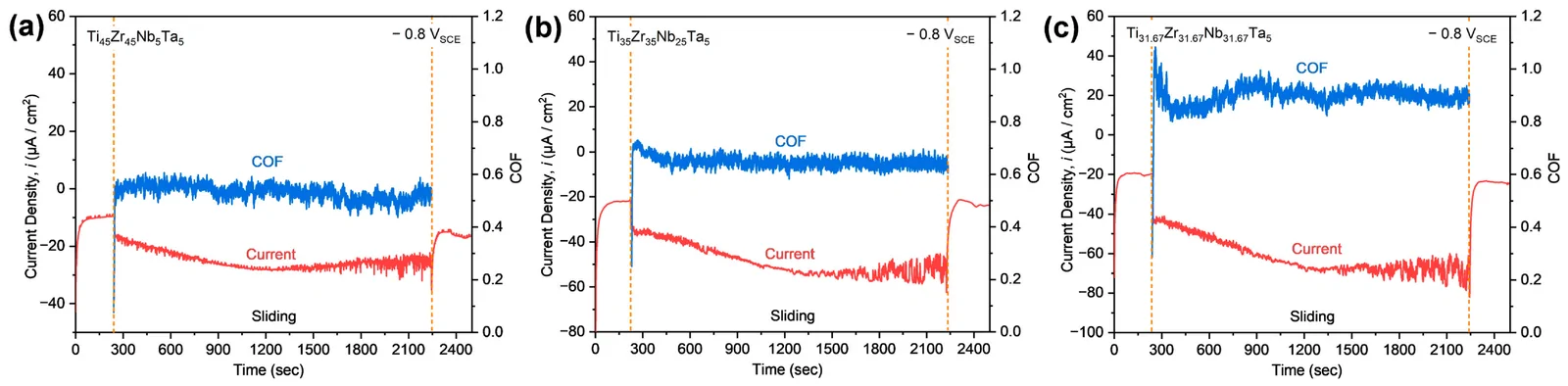
Current density and friction coefficient evolution of TiZrNbTa RMPEAs during tribocorrosion sliding at an applied cathodic potential of −0.8 V_SCE_: (**a**) Ti_45_Zr_45_Nb_5_Ta_5_; (**b**) Ti_35_Zr_35_Nb_25_Ta_5_; (**c**) Ti_31.67_Zr_31.67_Nb_31.67_Ta_5_.

The friction coefficients of the three alloys monitored during friction at the OCP potential are shown in Figure 4a–c. It can be seen that the friction coefficients of the three alloys remain stable during sliding. The average friction coefficients of Ti_45_Zr_45_Nb_5_Ta_5_, Ti_35_Zr_35_Nb_25_Ta_5_, and Ti_31.67_Zr_31.67_Nb_31.67_Ta_5_ alloys are 0.51 ± 0.05, 0.60 ± 0.04 and 0.78 ± 0.08, respectively. Under identical experimental conditions, the friction coefficient of metallic materials is mainly affected by surface roughness, surface lubrication and surface hardness. Owing to the identical surface treatment and passivated state of the specimens, the influence of surface roughness can be neglected. Therefore, the friction coefficient is primarily determined by the stability of the tribofilms formed on the tribocorrosion surfaces of the three alloys and by the tribocorrosion mechanism [48,49]. The tribocorrosion mechanisms of all tested alloys are discussed in the following section to analyze their effects on the friction coefficient. Under the applied cathodic potential condition, friction leads to an increase in cathodic current density for the three alloys (Figure 5a–c). This is attributed to the stirring effect of friction, which accelerates the cathodic reaction kinetics on the electrode surface. Furthermore, friction removes the residual passive film on the surface and exposes fresh metal, resulting in further acceleration of the cathodic reaction within the wear track region [49]. The residual current fluctuations under cathodic polarization (Figure 5) do not arise from anodic dissolution, which is suppressed at −0.8 V_SCE_, but mainly reflect the intermittent exposure of fresh metal areas and the entrainment and ejection of oxide debris (third-body effects) synchronized with the sliding cycles, as well as the perturbation of mass transfer in the cathodic reaction at the electrode surface caused by mechanical stirring. At cathodic potential, the average friction coefficients of Ti_45_Zr_45_Nb_5_Ta_5_, Ti_35_Zr_35_Nb_25_Ta_5_, and Ti_31.67_Zr_31.67_Nb_31.67_Ta_5_ alloys are 0.54 ± 0.05, 0.67 ± 0.05 and 0.89 ± 0.09, respectively. The cathodic potential suppresses both the anodic dissolution of the metal and oxide film formation, which may to some extent inhibit tribofilm formation and thus lead to a higher friction coefficient than that under OCP conditions.

### 3.4. Characterization of Tribocorroded Surface Morphology

**Figure 6:**
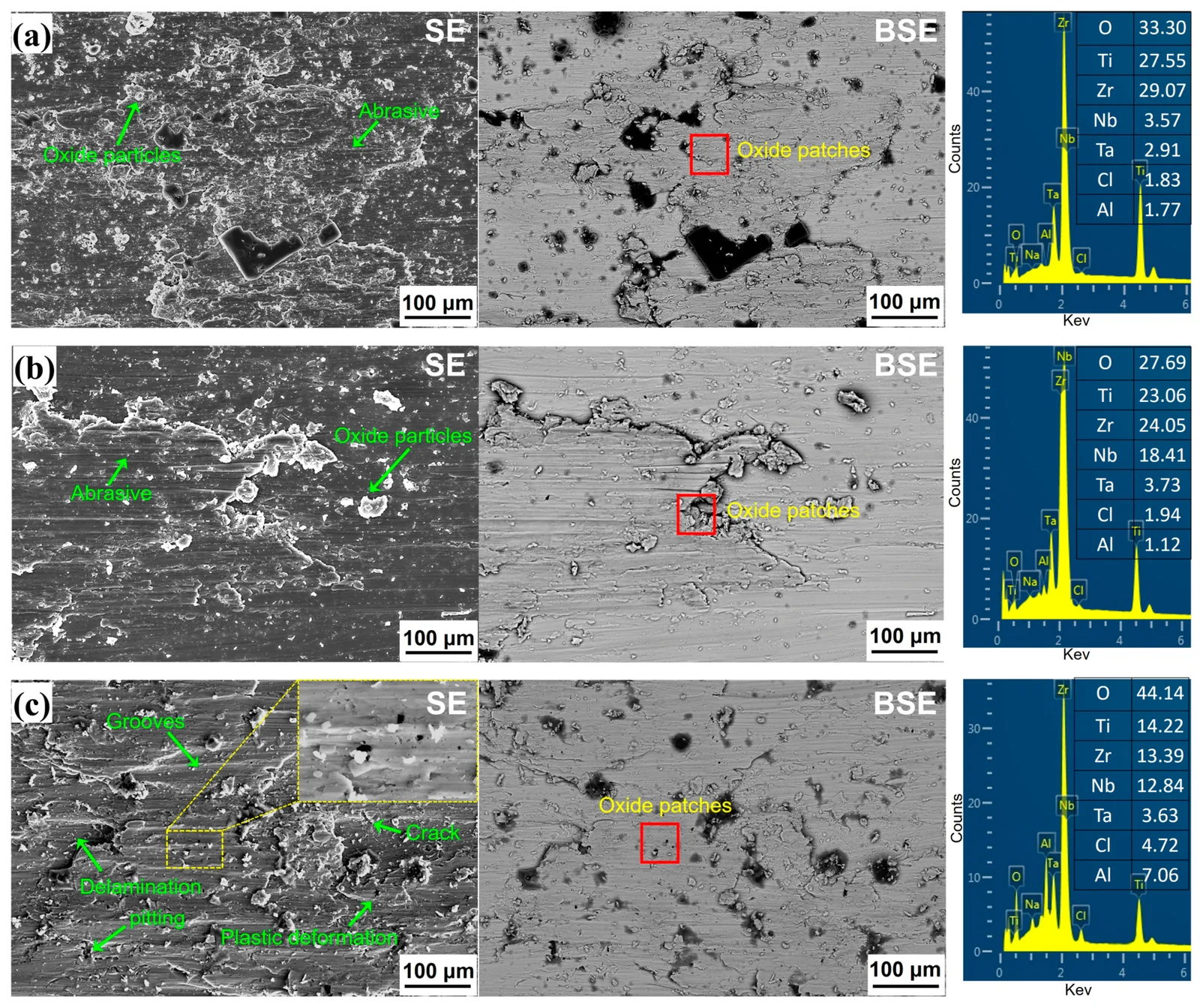
SEM, BSE, together with EDS spectra of tribocorrosion wear tracks on TiZrNbTa RMPEAs under OCP condition: (**a**) Ti_45_Zr_45_Nb_5_Ta_5_; (**b**) Ti_35_Zr_35_Nb_25_Ta_5_; (**c**) Ti_31.67_Zr_31.67_Nb_31.67_Ta_5_.

Figure 6 presents the SEM morphologies and corresponding back-scattered electron (BSE) images of tribocorrosion surfaces for the investigated alloys under OCP conditions. Abrasive wear features along the sliding direction can be observed on the tribocorrosion surface of the Ti_45_Zr_45_Nb_5_Ta_5_ alloy, which originate from micro-cutting induced by detached oxide particles (Figure 6a). Numerous island-like tribofilms are visible in the dark regions of its BSE image. The tribofilms are composed of oxides of each constituent element and contain a small amount of Al. The Al signal originates from minor material transfer caused by wear of the friction counterpart. The Ti_35_Zr_35_Nb_25_Ta_5_ alloy exhibits aggravated abrasive wear with abundant residual oxide particles on the surface. BSE and EDS results confirm that more discontinuous tribofilms form on the tribocorrosion surface (Figure 6b). Wider ploughing grooves and deteriorated abrasive wear appear on the tribocorrosion surface of the Ti_31.67_Zr_31.67_Nb_31.67_Ta_5_ alloy. Obvious material delamination, plastic deformation and microcrack propagation are also identified, and tiny pits are observed in local regions (Figure 6c). This phenomenon arises from friction-induced removal of the passive film, which accelerates chloride attack on the freshly exposed substrate surface. The coverage area and continuity of tribofilms on this alloy decrease. EDS measurements reveal a higher Al content in the tribofilms. This is attributed to severe wear of the friction counterpart and substantial transfer of counterpart materials onto the sample surface.

**Figure 7:**
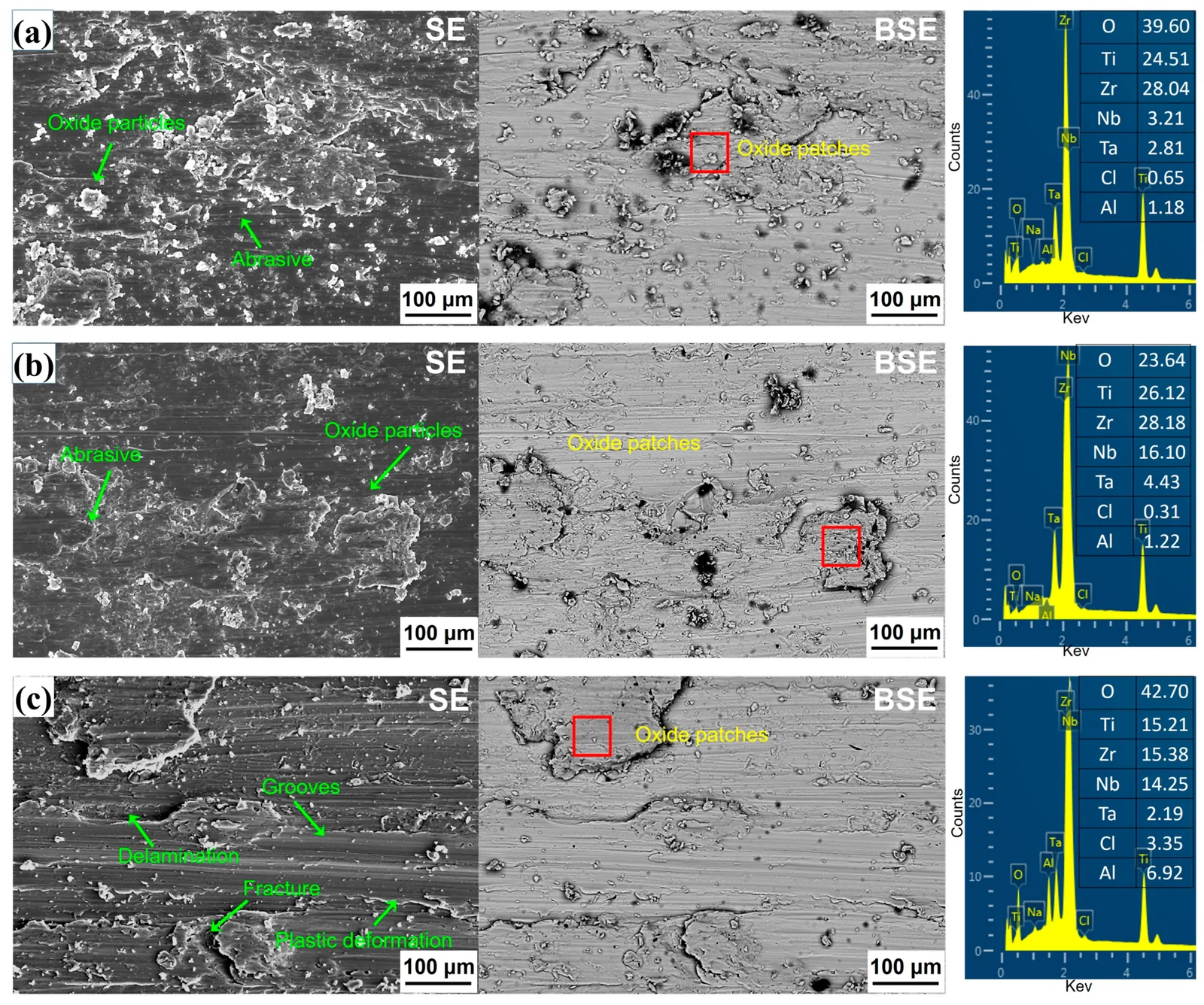
SEM, BSE, together with EDS spectra of tribocorrosion wear tracks on TiZrNbTa RMPEAs under −0.8 V_SCE_ cathodic potential: (**a**) Ti_45_Zr_45_Nb_5_Ta_5_; (**b**) Ti_35_Zr_35_Nb_25_Ta_5_; (**c**) Ti_31.67_Zr_31.67_Nb_31.67_Ta_5_.

When the cathodic potential is applied, the degree of abrasive wear on the tribocorrosion surfaces of Ti_45_Zr_45_Nb_5_Ta_5_ and Ti_35_Zr_35_Nb_25_Ta_5_ alloys exhibits no significant change. Obvious plastic deformation features emerge in local regions of the specimens. The continuity of the tribofilm decreases and local cracking and spalling can be observed, as shown in Figure 7a,b. The plastic deformation and delamination wear on the tribocorrosion surface of Ti_31.67_Zr_31.67_Nb_31.67_Ta_5_ alloy are further intensified, and prominent spalling of the tribofilm occurs. EDS analyses reveal a reduced Cl content on the tribocorrosion surface. This indicates that cathodic polarization suppresses chloride-induced surface corrosion. The above results demonstrate that cathodic potential aggravates surface wear damage for all tested alloys. The tribocorrosion surface morphologies confirm that the wear severity follows the order Ti_31.67_Zr_31.67_Nb_31.67_Ta_5_ > Ti_35_Zr_35_Nb_25_Ta_5_ > Ti_45_Zr_45_Nb_5_Ta_5_. It can therefore be concluded that increasing Nb content in TiZrNbTa alloys reduces their tribocorrosion resistance, and higher Nb content impairs the ability of the alloy to resist abrasive wear and delamination wear.

### 3.5. Analysis of the Tribocorrosion Rate

**Figure 8:**
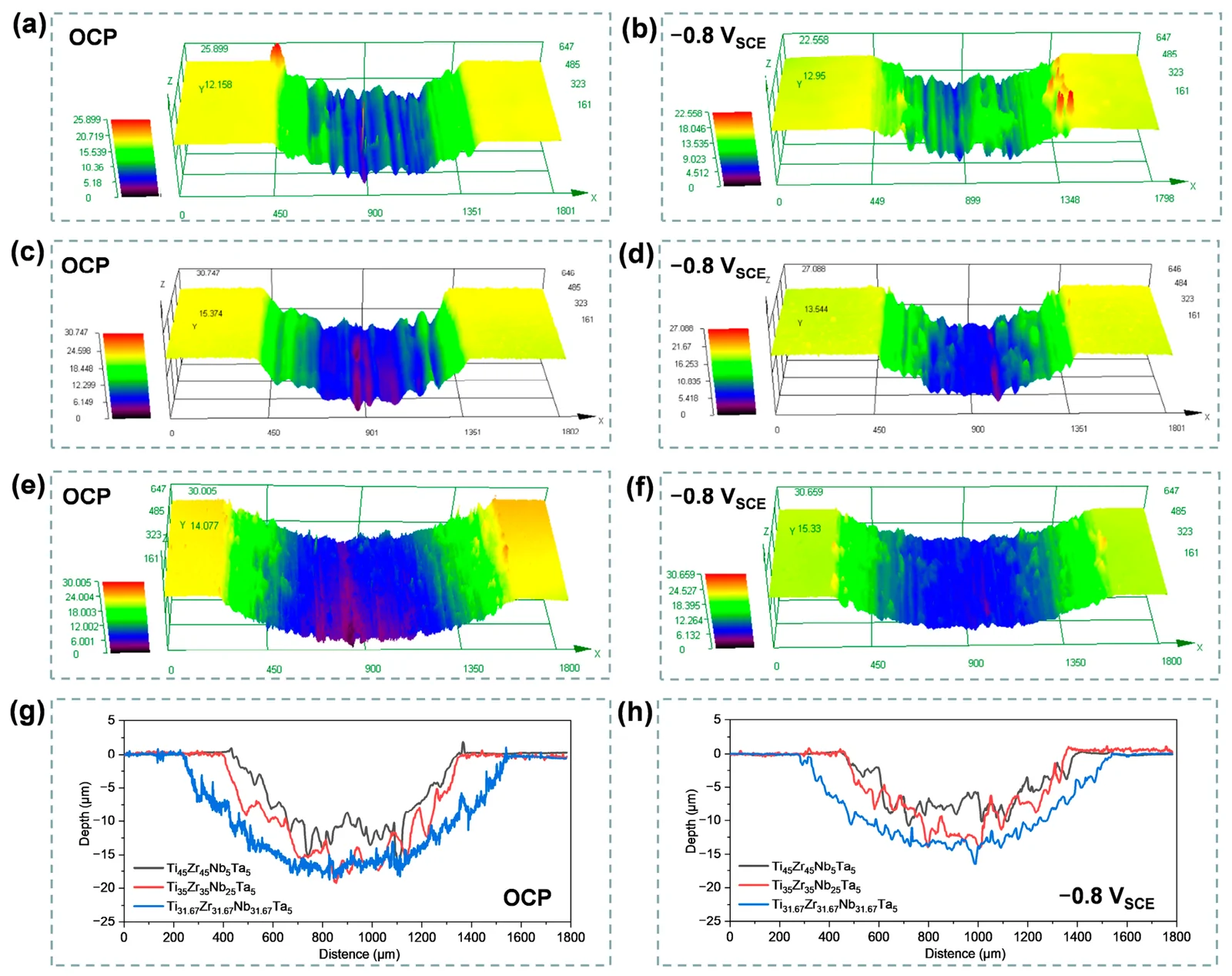
Three-dimensional topographies of wear tracks and corresponding cross-sectional profiles for TiZrNbTa RMPEAs under OCP and −0.8 V_SCE_ cathodic potential: (**a**,**b**) Ti_45_Zr_45_Nb_5_Ta_5_; (**c**,**d**) Ti_35_Zr_35_Nb_25_Ta_5_; (**e**,**f**) Ti_31.67_Zr_31.67_Nb_31.67_Ta_5_; (**g**) cross-sectional profiles under OCP; (**h**) cross-sectional profiles under −0.8 V_SCE_.

Figure 8 presents the three-dimensional morphologies and cross-sectional profiles of wear tracks measured for the three alloys under OCP and applied cathodic potential conditions. Under both open-circuit and cathodic potentials, the depth and width of wear tracks increase with rising Nb content in the alloys. Ti_31.67_Zr_31.67_Nb_31.67_Ta_5_ exhibits markedly severer wear than the other two alloys, which is associated with severe ploughing and delamination wear. The trend indicates that increasing Nb fraction weakens the protective capacity of the mechanically mixed layer formed within the wear track during sliding. A higher Nb content slows the repassivation kinetics after film rupture, which extends the duration of active dissolution and facilitates the initiation and propagation of surface cracks that drive delamination. According to the Archard equation, the total tribocorrosion rate can be expressed as [50]:

(3) $$k_{\mathrm{wt}}\;=\;\frac{\Delta V}{L\times N}$$

where $k_{\mathrm{wt}}$ is the total tribocorrosion rate, ΔV is the total wear volume (mm^3^), L is the sliding distance (m), and N is the normal load (N). The calculated tribocorrosion rates of Ti_45_Zr_45_Nb_5_Ta_5_, Ti_35_Zr_35_Nb_25_Ta_5_ and Ti_31.67_Zr_31.67_Nb_31.67_Ta_5_ alloys under open-circuit conditions are (3.75 ± 0.11) × 10^−3^, (5.77 ± 0.17) × 10^−3^ and (10.27 ± 0.30) × 10^−3^ mm^3^·N^−1^·m^−1^, respectively. Under cathodic potential, the values are (2.97 ± 0.09) × 10^−3^, (4.23 ± 0.13) × 10^−3^ and (7.26 ± 0.22) × 10^−3^ mm^3^·N^−1^·m^−1^, respectively. For all three compositions, the cathodic condition yields a lower wear rate than OCP, which further verifies the coupling effect between mechanical damage and electrochemical corrosion in tribocorrosion. At −0.8 V_SCE_, anodic dissolution is largely inhibited and the contribution of corrosion-accelerated wear is eliminated. The reduced tribocorrosion rate under cathodic potential is attributed to the corrosion suppression by cathodic polarization, where only pure mechanical wear dominates. 

### 3.6. XPS Characterization of Tribocorroded Surfaces

**Figure 9:**
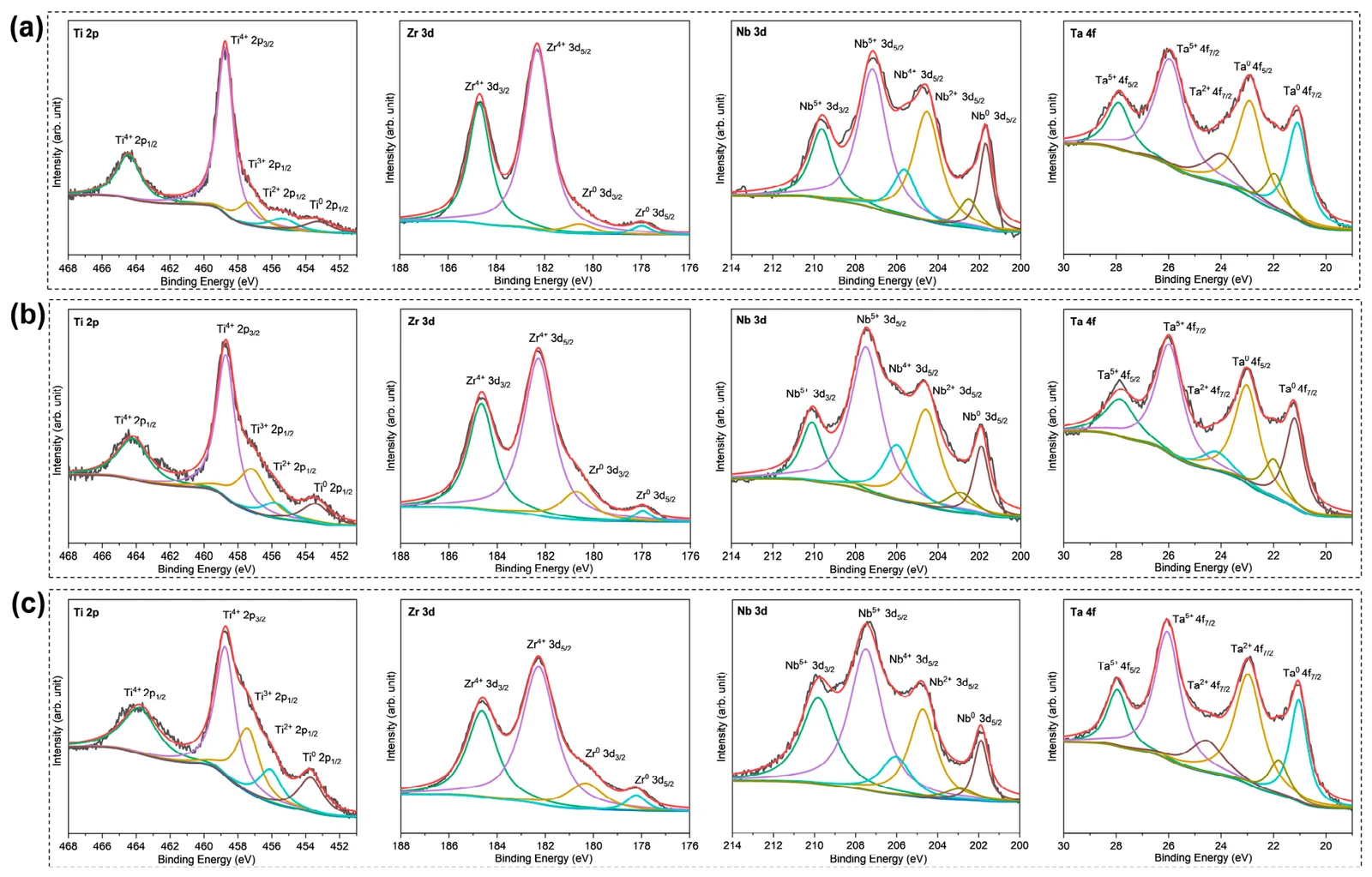
High-resolution XPS spectra (Ti 2p, Zr 3d, Nb 3d and Ta 4f) of the tribocorroded surfaces on TiZrNbTa RMPEAs after 10 s immersion following sliding under OCP: (**a**) Ti_45_Zr_45_Nb_5_Ta_5_; (**b**) Ti_35_Zr_35_Nb_25_Ta_5_; (**c**) Ti_31.67_Zr_31.67_Nb_31.67_Ta_5_.

**Figure 10:**
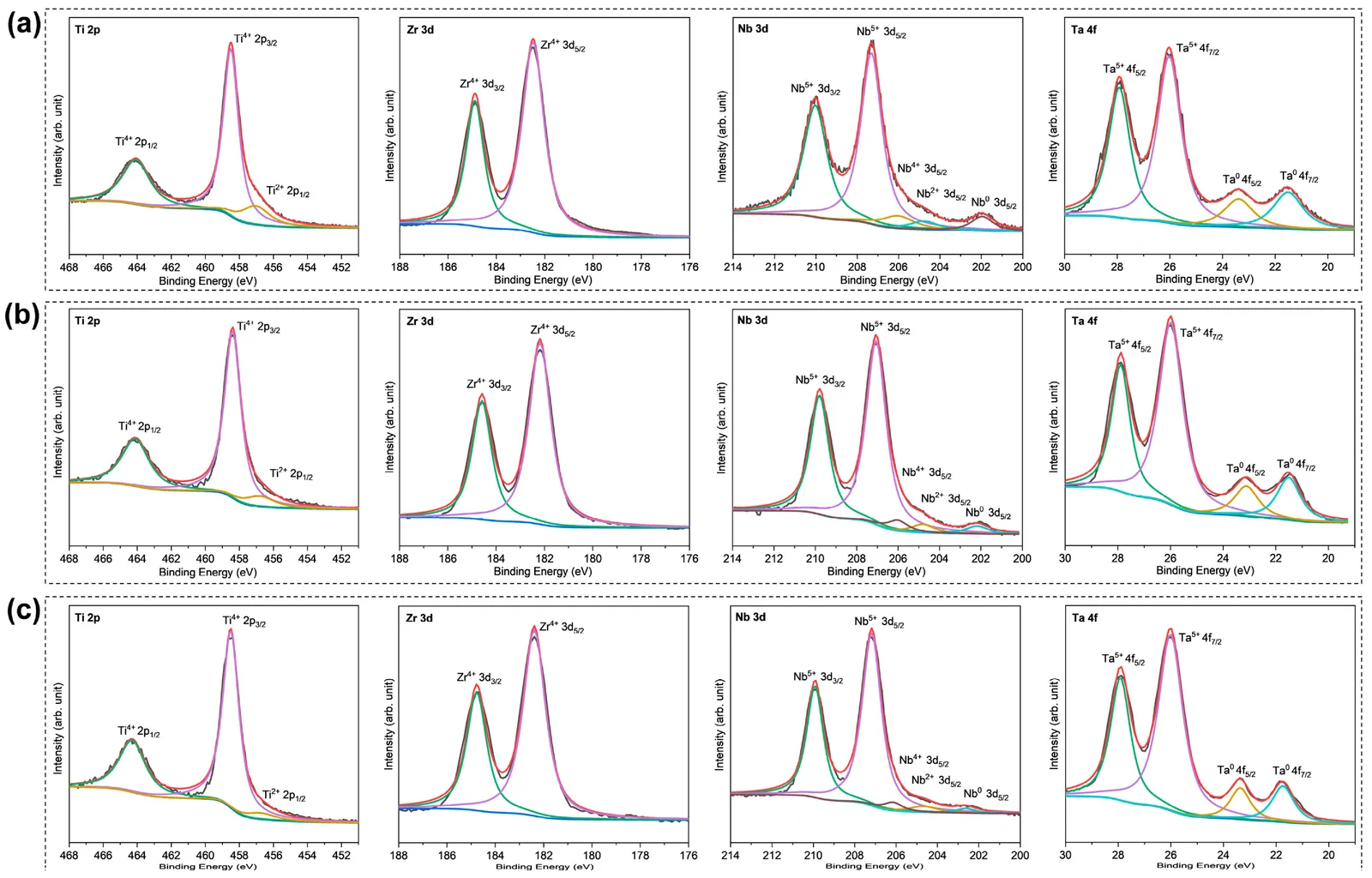
High-resolution XPS spectra (Ti 2p, Zr 3d, Nb 3d and Ta 4f) of the tribocorroded surfaces on TiZrNbTa RMPEAs after 2 h immersion following sliding under OCP: (**a**) Ti_45_Zr_45_Nb_5_Ta_5_; (**b**) Ti_35_Zr_35_Nb_25_Ta_5_; (**c**) Ti_31.67_Zr_31.67_Nb_31.67_Ta_5_.

Figure 9 and Figure 10 show the high-resolution XPS spectra of surface films on three alloys after tribocorrosion under OCP, followed by immersion in the solution for 10 s and 2 h, respectively. As shown in Figure 9, Ti and Zr preferentially form high-valence oxides at the initial stage of repassivation. In the high-resolution Ti 2p spectrum, the spin–orbit split doublet of Ti^4+^ dominates, with only weak signals of Ti^3+^, Ti^2+^ and metallic Ti^0^ detected on the low-binding-energy side. Similarly, the Zr 3d spectrum is dominated by the Zr^4+^ doublet, accompanied by minor Zr^2+^ and Zr^0^ species. These results confirm that Ti and Zr can be rapidly oxidized to thermodynamically stable TiO_2_ and ZrO_2_ at the early nucleation stage of the passive film. Among the three alloy systems, the relative content of TiO_2_ and ZrO_2_ is the highest on the Ti_45_Zr_45_Nb_5_Ta_5_ surface. With the increase in Nb fraction and the corresponding reduction in Ti/Zr content in the alloy, the proportion of intermediate-valence and metallic species of Ti and Zr rises, indicating insufficient oxidation. The Nb 3d spectrum is dominated by Nb^5+^ peaks, and the Ta 4f spectrum is dominated by Ta^5+^ peaks. Nevertheless, the relative intensities of the intermediate-valence and metallic characteristic peaks of Nb and Ta are markedly higher than those of Ti and Zr, indicating the slower oxidation rate and retarded growth of the passive film for Nb and Ta. Further comparison among the three alloys reveals that the relative fraction of Nb^5+^ increases and the peak intensities of intermediate-valence and metallic Nb species decrease with increasing Nb content, while no obvious changes are observed in the characteristic peaks of Ta at different valence states. The intensity of metallic peaks measured by XPS can qualitatively characterize the equivalent oxide film thickness and surface coverage of each element. A stronger metallic signal indicates a thinner corresponding oxide layer or poorer coverage integrity. The metallic signals of Ti and Zr are nearly undetectable, whereas relatively strong metallic signals remain for Nb and Ta. This result demonstrates that Ti and Zr undergo preferential oxidation at the initial repassivation stage and govern the rapid nucleation and growth of the oxide layer after the breakdown of the original passive film. Alloys with higher Ti and Zr contents possess faster repassivation rates on the fresh bare metallic surface, which is consistent with the violent fluctuation of potential curves during tribocorrosion under OCP conditions and the rapid potential recovery after tribocorrosion ceases.

As shown in Figure 10, the passive film continuously grows and stabilizes after 2 h of immersion. The intermediate-valence and metallic species of all elements transform into high-valence states. The signals of Ti^0^ and Zr^0^ completely disappear, and Ti and Zr mainly exist in the forms of Ti^4+^ and Zr^4+^. By contrast, certain metallic and intermediate-valence species are still detected in the Nb and Ta spectra, indicating that their oxidation and film formation processes remain slower than those of Ti and Zr. As the Nb content in the alloy increases, the relative fraction of high-valence Nb^5+^ oxides in the passive film rises further. These results reveal that the passive film gradually evolves into a mixed oxide film consisting of high-valence oxides of the four elements after long-term immersion, and its compositional evolution is directly related to the elemental ratio of the alloy.

## 4. Discussion

A substantial increase in the Nb content of the alloy reduces its repassivation rate, which originates from the corresponding decrease in Ti and Zr fractions. Upon exposure of the freshly generated alloy surface to the aqueous solution, the constituent elements dissolve anodically to release metal ions, while hydroxide ions adsorb on the bare metal surface via a partial charge transfer process to form a two-dimensional hydroxide adlayer as the precursor of the passive film, which subsequently precipitates as metal hydroxide and finally dehydrates into the oxide film. The relevant reactions are presented below [51].

(4) $$Me\to Me^{n+}+ne^{-}$$

(5) $$Me+OH^{-}\to Me-OH_{ads}^{\left(\gamma -\right)}+(1-\gamma )e^{-}$$

(6) $$Me+nOH^{-}\to Me(OH{)}_{n}+ne^{-}$$

(7) $$Me^{n+}+nOH^{-}\to Me(OH{)}_{n}↓$$

(8) $$2Me(OH{)}_{n}\to Me_{2}O_{n}+nH_{2}O$$

In the above reactions, *Me* denotes each constituent metallic element of the alloy (i.e., Ti, Zr, Nb, and Ta). Ti and Zr are passivity-promoting elements, which preferentially adsorb oxygen or hydroxide ions to form oxides after dissolution. According to thermodynamic calculations [52], the equilibrium electrode potentials for the formation of high-valence oxides of each element are listed as follows: Ti → TiO_2_ (−1.490 V), Zr → ZrO_2_ (−1.887 V), Nb → Nb_2_O_5_ (−1.015 V), Ta → Ta_2_O_5_ (−1.166 V). The equilibrium potentials of these redox reactions (vs. the standard hydrogen electrode) reveal that Ti and Zr exhibit more negative values. Thermodynamically, they are more prone to oxidation, preferentially forming stable surface oxide layers and governing the rapid nucleation and growth of oxides. Accordingly, alloys with higher Ti and Zr contents (e.g., Ti_45_Zr_45_Nb_5_Ta_5_ alloy) exhibit a faster rise in OCP during passive film growth. The beneficial effect of Nb addition on the stability of passive films has been demonstrated in various Ti-based MPEAs, such as TiZrNb [53], TiZrHfNb [54,55], TiZrNbV [56] and TiVZrNbAl [57]. The underlying mechanism is mainly attributed to the similar ionic radii and electronic configurations of Nb^5+^ and Ti^4+^. Nb can preferentially dissolve into the TiO_2_ oxide and substitute for Ti^4+^, forming a substitutional defect with an effective positive charge, $Nb_{\mathrm{Ti}}^{\text{·}}$. According to the Kröger–Vink defect chemistry [58,59], the effective charge of $Nb_{\mathrm{Ti}}^{\text{·}}$ is compensated by the formation of Ti vacancies ($V_{\mathrm{Ti}}^{‘’’’}$), and the two species further associate into electrically neutral defect complexes, thereby reducing the concentration of mobile point defects, such as oxygen vacancies, within the passive film. Therefore, increasing the Nb addition can further reduce the concentration of oxygen vacancy defects in the passive film, thereby suppressing the adsorption of Cl^−^ and decreasing the inward diffusion rate of cation vacancies generated by the Schottky pair reaction [60], which enables the passive film to maintain its high protective performance and stability.

In tribocorrosion systems, the total measured material loss is not simply the sum of material losses induced by pure electrochemical corrosion and pure wear. For certain alloys, the synergistic effect between wear and corrosion can lead to much more severe material removal than that caused by pure corrosion or pure wear alone [61]. The synergistic effect of tribocorrosion can be quantified using the following equation.

(9) $$W_{t}=W_{f}+W_{c}+\Delta W_{s}$$

where $W_{t}$ is the total material loss of the alloy, $W_{f}$ is the pure mechanical wear component, $W_{c}$ is the pure corrosion component, and $\Delta W_{s}$ represents the material loss induced by the synergistic effect. In this equation, $\Delta W_{s}$ is the sum of wear-accelerated corrosion ($\Delta W_{cf}$) and corrosion-accelerated wear ($\Delta W_{fc}$). Therefore, $\Delta W_{s}$ is expressed as:

(10) $$\Delta W_{s}=\Delta W_{fc}+\Delta W_{cf}$$

By measuring the total tribocorrosion volume loss under open-circuit conditions and cathodic potentials, the terms $W_{t}$ and $W_{f}$ in Equation (9) can be obtained, respectively. According to Faraday’s law, $W_{c}$ and $\Delta W_{cf}$ can be calculated using Equations (11) and (12), respectively [61].

(11) $$W_{c}=\frac{ItM}{nF\rho }$$

(12) $$\Delta W_{cf}=\frac{I_{f}tM}{nF\rho }$$

where *I* is the current under static conditions (A). Under OCP conditions, $I_{f}$ denotes the difference in corrosion current between static and sliding states. *t* is the sliding time (s), F is Faraday’s constant (C·mol^−1^), *ρ* is the alloy density (g·cm^−3^), M is the molar mass of the alloy, and *n* is the number of charges transferred in the oxidation reaction. M is calculated by $\sum x_{i}M_{i}$, where $x_{i}$ is the atomic fraction of each element and $M_{i}$ is its relative atomic mass. Subsequently, the corrosion-induced wear component ($\Delta W_{fc}$) can be obtained by subtracting $\Delta W_{cf}$ from $\Delta W_{s}$ in Equation (10). The individual tribocorrosion components of Ti_45_Zr_45_Nb_5_Ta_5_, Ti_35_Zr_35_Nb_25_Ta_5_ and Ti_31.67_Zr_31.67_Nb_31.67_Ta_5_ alloys were calculated using the tribocorrosion component decomposition equations, and the results are listed in Table 2. Under open-circuit conditions, the fractions of pure mechanical wear component $W_{f}$ in the total tribocorrosion loss are 79.11%, 73.23% and 70.72% for Ti_45_Zr_45_Nb_5_Ta_5_, Ti_35_Zr_35_Nb_25_Ta_5_ and Ti_31.67_Zr_31.67_Nb_31.67_Ta_5_ alloys, respectively, demonstrating that material removal is dominated by pure mechanical friction. Meanwhile, the corresponding fractions of synergistic component $\Delta W_{s}$ are 20.89%, 26.77% and 29.28%. It can be observed from the table that the $\Delta W_{fc}$ term is markedly larger than $\Delta W_{cf}$ for all tested alloys, indicating that corrosion-accelerated wear dominates the synergistic interaction during tribocorrosion. By contrast, $W_{c}$ and $\Delta W_{cf}$ account for very small proportions of the total material loss. Therefore, mechanical wear governs the material loss of these alloys during tribocorrosion. At −0.8 V_SCE_, the current density of all alloys stays cathodic during sliding. Friction accelerates the cathodic process and raises cathodic current density (Figure 5), and no anodic peaks appear in the curves, demonstrating that cathodic reactions govern the electrode process and anodic dissolution of newly exposed surfaces is suppressed. Even at OCP, $W_{c}$ and $\Delta W_{cf}$ make up less than 0.2% and 1% of the total loss, respectively (Table 2), and these fractions decline further at −0.8 V_SCE_. This potential suppresses Cl ion adsorption, resulting in lower Cl content on worn surfaces without observable pitting or corrosion-initiated cracks (Figure 7). Furthermore, many previous studies have used −0.8 V_SCE_ to evaluate pure mechanical wear of Ti-based alloys [62,63,64,65]. Accordingly, corrosion contributes negligibly to material loss at this cathodic potential.

Ti_45_Zr_45_Nb_5_Ta_5_ alloy exhibits a high hardness of 320 ± 10 HV and forms abundant tribofilms on the tribocorroded surface, which are responsible for its low friction coefficient and low tribocorrosion rate. Abrasive wear dominates its wear mechanism, with no evidence of crack propagation or delamination. During friction, fine spalled oxide particles impose mild micro-cutting on the contact surface and induce slight abrasive wear. The tiny wear debris tends to accumulate and agglomerate at the friction interface, and is continuously compressed by the counterbody to form a continuous tribofilm [48,66]. For Ti_35_Zr_35_Nb_25_Ta_5_ alloy with a hardness of 280 ± 12 HV, the degree of abrasive wear increases. Distinct abrasive wear features can be observed on the tribofilm surface, indicating that tribofilms can mitigate micro-cutting of the alloy substrate by abrasive particles to a certain extent. Nevertheless, the reduced substrate hardness compared with Ti_45_Zr_45_Nb_5_Ta_5_ promotes plastic deformation. The locally formed tribofilms cannot accommodate the coordinated deformation of the matrix and thus lose stability. The fractured and spalled tribofilm fragments participate in three-body wear, aggravating abrasive wear and increasing the friction coefficient. Severe ploughing, delamination and plastic deformation occur on the tribocorroded surface of Ti_31.67_Zr_31.67_Nb_31.67_Ta_5_ alloy, which is related to its low hardness (230 ± 10 HV). Severe surface plastic deformation and ploughing during friction greatly hinder the stable formation of tribofilms, leading to a high friction coefficient and high material loss rate.

**Figure 11:**
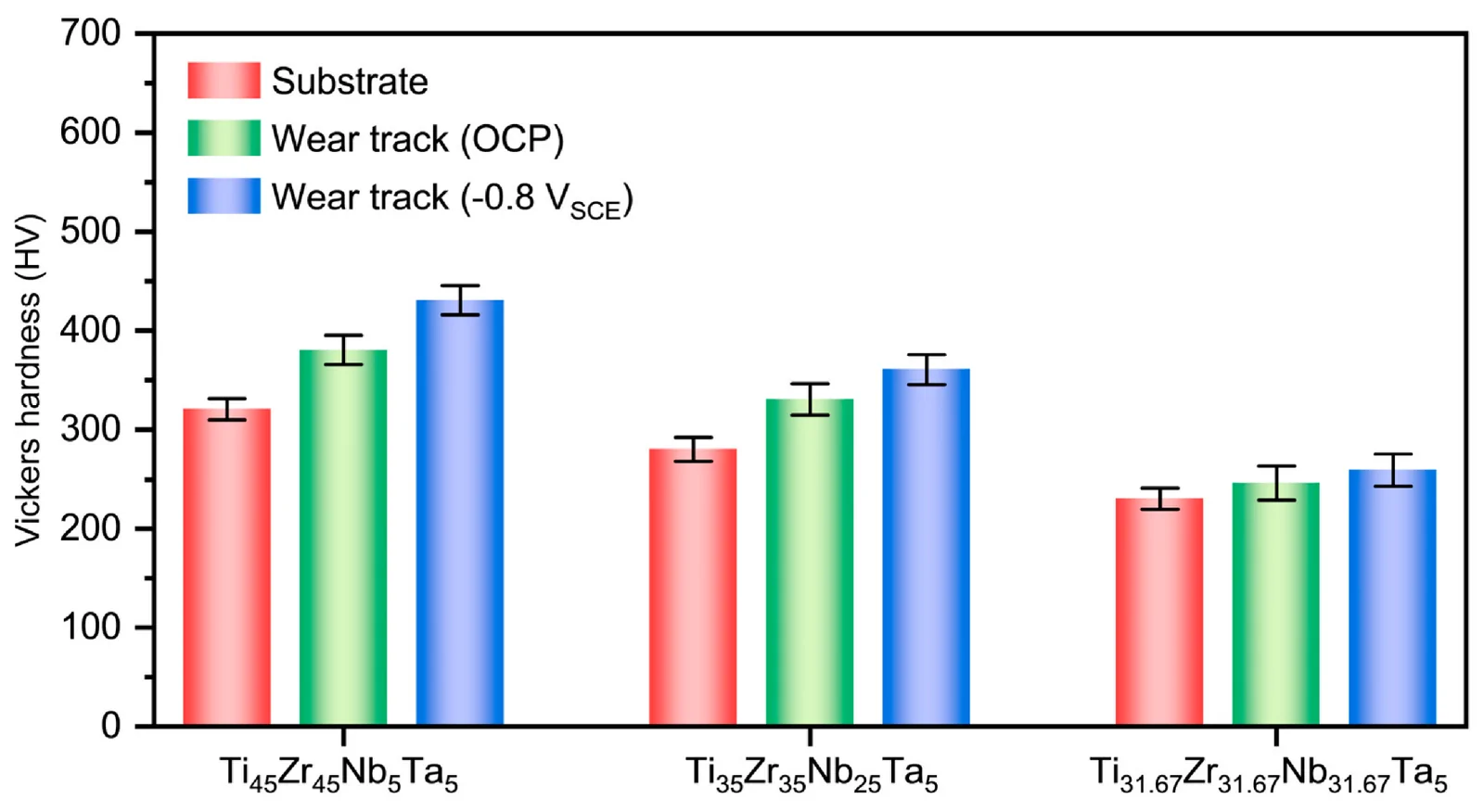
Vickers hardness of the substrate and wear tracks for TiZrNbTa RMPEAs after tribocorrosion under OCP and −0.8 V_SCE_.

**Figure 12:**
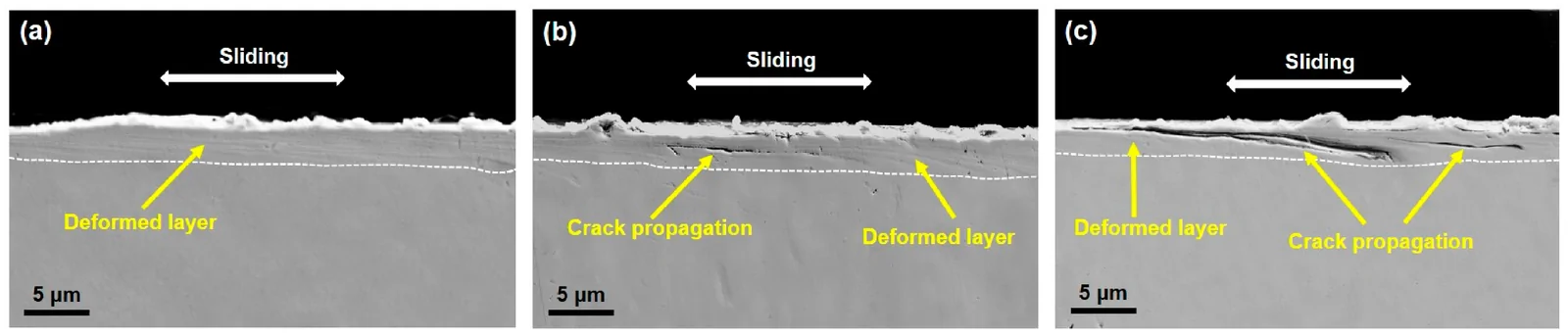
Cross-sectional SEM micrographs showing subsurface deformed layer and crack propagation of wear tracks: (**a**) Ti_45_Zr_45_Nb_5_Ta_5_; (**b**) Ti_35_Zr_35_Nb_25_Ta_5_; (**c**) Ti_31.67_Zr_31.67_Nb_31.67_Ta_5_.

Plastic deformation occurs in the near-surface region of materials under frictional shear stress. Dislocation motion and rearrangement during deformation refine grains and induce work hardening in the surface layer [67]. Figure 11 shows the variation in surface hardness of the three alloys after tribocorrosion at different potentials. The degree of work hardening under cathodic potential is markedly higher than that under OCP. During deformation, dislocations accumulate in the shallow subsurface and form pile-ups, resulting in stress concentration and subsequent crack nucleation and propagation. Shallow deformation features can be identified on Ti_45_Zr_45_Nb_5_Ta_5_ alloy, while no fatigue cracks are detected (Figure 12a). Its deformed layer remains stable and produces prominent work hardening (Figure 11). Benefiting from higher substrate hardness, Ti_35_Zr_35_Nb_25_Ta_5_ alloy exhibits milder subsurface deformation than Ti_31.67_Zr_31.67_Nb_31.67_Ta_5_, which slows the nucleation and propagation of microcracks and leads to slight delamination wear (Figure 12b). Distinct microcrack propagation is observed in the shallow subsurface of the Ti_31.67_Zr_31.67_Nb_31.67_Ta_5_ alloy (Figure 12c). This weakens the work-hardening effect, and extensive material removal prevents the stable retention of the work-hardened layer.

**Figure 13:**
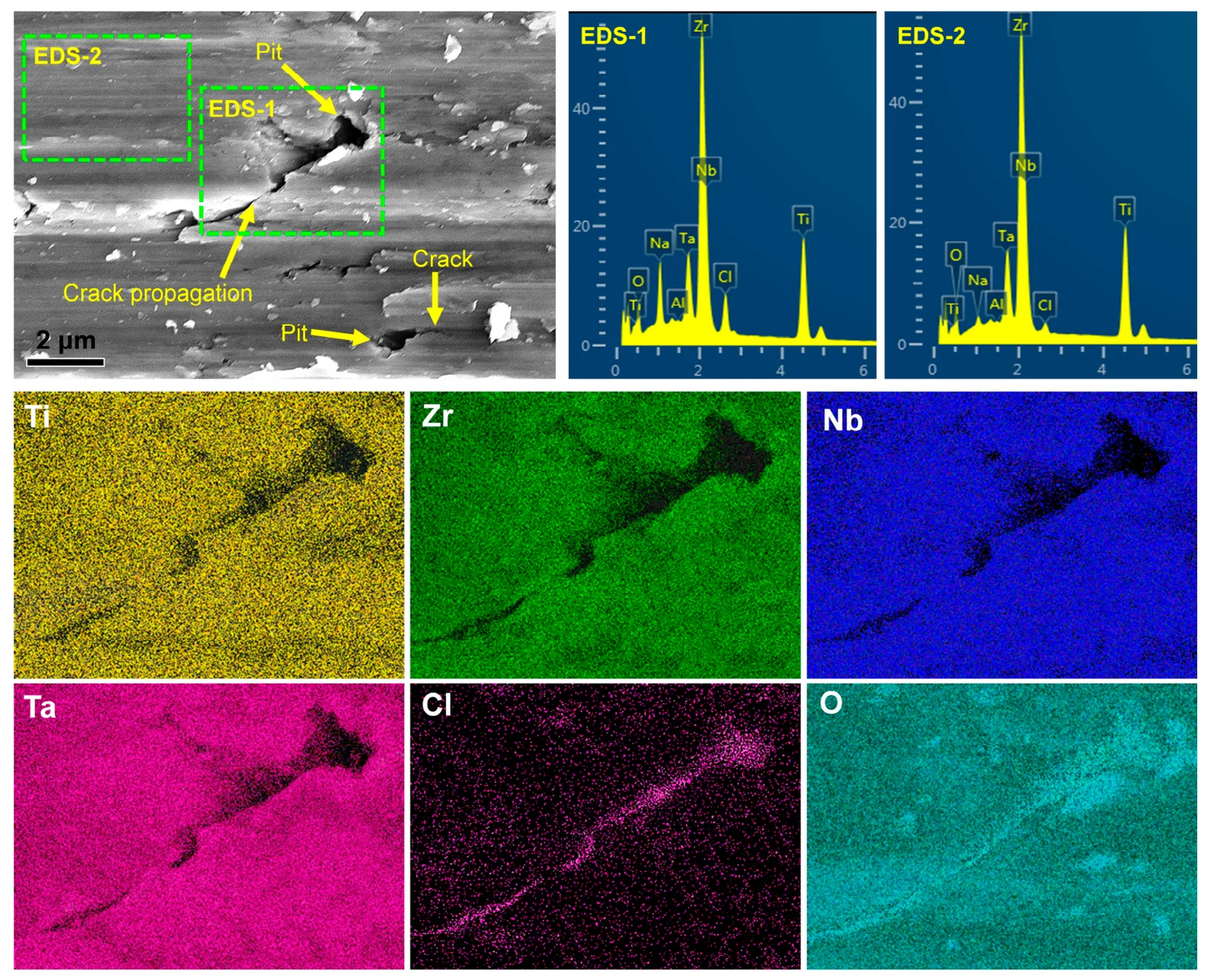
Surface SEM morphology, EDS point spectra and elemental mapping of tribocorroded Ti_31.67_Zr_31.67_Nb_31.67_Ta_5_ alloy showing pits, cracks and elemental distribution.

Ti_31.67_Zr_31.67_Nb_31.67_Ta_5_ and Ti_35_Zr_35_Nb_25_Ta_5_ alloys possess dendritic microstructures with obvious intergranular elemental segregation. Such compositional heterogeneity leads to differences in mechanical properties between dendritic and intergranular regions [68]. Under friction, this microstructural inhomogeneity causes incompatible deformation and triggers local stress concentration for crack initiation. The reduced work hardening under OCP (Figure 11) originates from the synergistic interaction between friction and corrosion [69,70]. Tribocorrosion component calculations reveal that corrosion-accelerated wear accounts for a larger proportion of total material loss for Ti_31.67_Zr_31.67_Nb_31.67_Ta_5_ alloy. Severe plastic deformation on its surface facilitates microcrack nucleation, and corrosive chloride ions further accelerate crack propagation [49]. Pitting pits generally act as stress concentration sites during plastic deformation, which initiate cracks and promote their further growth [49,71]. As shown in Figure 13, microcrack propagation originating from pitting pits is observed on the tribocorroded surface of Ti_31.67_Zr_31.67_Nb_31.67_Ta_5_ alloy. EDS analysis demonstrates obvious enrichment of Cl and O elements inside the pits and along crack propagation paths, accompanied by depletion of matrix elements. This phenomenon indicates that chloride attack induces corrosion dissolution within pits and cracks. In summary, increasing Nb content reduces alloy hardness and aggravates elemental segregation. Localized pitting accelerates crack nucleation and propagation during plastic deformation, suppresses the development of work hardening, and consequently intensifies corrosion-accelerated wear.

This series adopts a design with equal proportional reduction in Ti and Zr and simultaneous increase in Nb content, following a well-established strategy for investigating Nb alloying effects in Ti/Zr-based alloys. Ji et al. [53] employed this same modulation scheme in the biomedical Ti_(100−x)/2_Zr_(100−x)/2_Nb_x_(x = 0–33.33 at.%). Nevertheless, this design carries an inherent limitation: the increase in Nb is accompanied by a concurrent drop in Ti and Zr fractions. Variations in hardness, repassivation and tribocorrosion performance arise from the overall change in the Nb/(Ti + Zr) ratio and cannot be directly attributed to the isolated effect of Nb. Accordingly, this work only establishes performance evolution trends across different compositions. The role of Nb is indirectly supported by XPS evidence of preferential oxidation and defect chemistry mechanisms. Future designs that fix the total content of Ti and Zr, with Ta serving as a compensating element, will enable quantitative decoupling of the independent contribution of Nb.

## 5. Conclusions

In this work, three Ti_(95−x)/2_Zr_(95−x)/2_Nb_x_Ta_5_ (x = 5, 25, 31.67 at.%) RMPEAs with controlled Nb content were fabricated by arc melting, and the effect of Nb content on their microstructure, passive film characteristics and tribocorrosion behavior in NaCl solution was systematically investigated. The main conclusions are as follows: 

(1) Three as-cast TiZrNbTa RMPEAs exhibit single-phase BCC solid-solution structures. Increasing Nb content refines grains but intensifies segregation, with Nb and Ta enriched in dendrites and Ti and Zr concentrated in interdendritic regions. The lattice constant decreases, and microhardness declines from 320 ± 10 HV to 230 ± 10 HV. 

(2) The *i*_corr_ and *i*_pass_ decrease with increasing Nb content, demonstrating that Nb improves the stability of the passive film. Under tribocorrosion conditions, sliding markedly increases the *i*_corr_ and shifts the *E*_corr_ negatively. In addition, the resistance of the alloy to friction-induced localized accelerated dissolution is enhanced as Nb content rises. 

(3) The repassivation rate of the alloys decreases with increasing Nb content. The rapid nucleation and growth of the passive film on freshly exposed surfaces are dominated by the preferential oxidation of Ti and Zr, while the oxidation of Nb and Ta are relatively sluggish.

(4) As the Nb content increases, the wear mechanism of the alloy changes from mild abrasive wear accompanied by abundant continuous tribofilms to severe ploughing, delamination, plastic deformation and microcrack propagation, and both the friction coefficient and the tribocorrosion rate increase accordingly.

(5) Pure mechanical wear dominates total material loss, while corrosion-accelerated wear is the dominant synergistic component and markedly exceeds wear-accelerated corrosion. This synergistic proportion increases with rising Nb content, indicating that higher Nb alloying enhances the acceleration of wear by corrosion.

(6) Increased Nb content lowers alloy hardness and worsens elemental segregation, readily causing local stress concentration under severe plastic deformation. Localized pits then act as stress raisers, triggering microcrack nucleation, while chloride ions further drive crack propagation, resulting in severe delamination wear. These effects suppress surface work hardening and intensify corrosion-accelerated wear, ultimately degrading the tribocorrosion resistance of TiZrNbTa RMPEAs.

## Figures and Tables

**Table 1:** EDS compositions (at.%) of the dendritic region (DR) and interdendritic region (IDRs) of the as-cast TiZrNbTa RMPEAs. DR and IDR denote the Nb/Ta-rich primary dendrite arms that solidified first and the Ti/Zr-rich regions between dendrite arms that solidified last, respectively.

Elements	Ti	Zr	Nb	Ta	Ti	Zr	Nb	Ta
	Dendritic				Interdendritic			
Ti_45_Zr_45_Nb_5_Ta_5_	44.59 ± 0.62	44.16 ± 0.58	5.54 ± 0.31	5.70 ± 0.28	45.11 ± 0.55	45.04 ± 0.61	5.05 ± 0.27	4.80 ± 0.24
Ti_35_Zr_35_Nb_25_Ta_5_	32.80 ± 0.71	29.44 ± 0.66	30.50 ± 0.72	7.26 ± 0.35	37.20 ± 0.68	39.30 ± 0.74	19.35 ± 0.58	3.15 ± 0.22
Ti_31.67_Zr_31.67_Nb_31.67_Ta_5_	29.47 ± 0.77	24.35 ± 0.69	38.40 ± 0.81	7.78 ± 0.38	35.10 ± 0.72	36.47 ± 0.78	24.90 ± 0.63	3.53 ± 0.25

**Table 2:** Calculated tribocorrosion volume components for TiZrNbTa RMPEAs. All components are annual volume losses in mm^3^; the multiplicative scaling factor shown in each column header (×10^2^ or ×10^−3^) must be applied to the tabulated values. The decomposition satisfies *W*_t_ = *W*_f_ + *W*_c_ + Δ*W*_cf_ + Δ*W*_fc_ for each alloy.

Materials	*W*_t_ (×10^2^ mm^3^)	*W*_f_ (×10^2^ mm^3^)	*W*_c_ (×10^−3^ mm^3^)	Δ*W*_cf_ (×10^2^ mm^3^)	Δ*W*_fc_ (×10^2^ mm^3^)
Ti_45_Zr_45_Nb_5_Ta_5_	11.244 ± 0.337	8.895 ± 0.267	0.185 ± 0.019	0.096 ± 0.005	2.253 ± 0.068
Ti_35_Zr_35_Nb_25_Ta_5_	17.313 ± 0.519	12.678 ± 0.380	0.062 ± 0.006	0.152 ± 0.008	4.483 ± 0.134
Ti_31.67_Zr_31.67_Nb_31.67_Ta_5_	30.809 ± 0.924	21.789 ± 0.654	0.023 ± 0.002	0.289 ± 0.014	8.731 ± 0.262

## Data Availability

The original contributions presented in this study are included in the article. Further inquiries can be directed to the corresponding author.
